# Splicing as an integrative layer linking cell cycle regulation, epigenetic dynamics, and environmental responses in plants

**DOI:** 10.3389/fpls.2026.1910340

**Published:** 2026-08-25

**Authors:** Geovanna Vitória Olimpio, Bruna Gino de Araújo-Lopes, Marcelo de Oliveira Gigier, Adriana Silva Hemerly

**Affiliations:** Laboratório de Biologia Molecular de Plantas, Instituto de Bioquímica Médica, Universidade Federal do Rio de Janeiro, Rio de Janeiro, Brazil

**Keywords:** cell cycle, chromatin remodeling, DNA damage, plant stress responses, spliceosome, splicing

## Abstract

Plants continuously adjust growth and development in response to environmental fluctuations through mechanisms that coordinate gene expression and cell cycle progression. Advances in our understanding of these regulatory pathways support a model in which they function as interconnected layers that fine-tune cell cycle progression according to developmental and environmental signals. Alternative splicing (AS) has emerged as a major regulatory layer in these processes, generating transcript diversity and modulating the activity of genes involved in developmental transitions and stress responses. Increasing evidence indicates that several spliceosomal components and splicing regulators are also connected to cell cycle control, influencing the expression, processing, or activity of key cell cycle regulators. In parallel, chromatin modifications and chromatin-associated factors shape transcriptional activity and splice-site selection, providing a mechanistic link between environmental perception and RNA processing. Here, we review recent progress in elucidating the mechanisms underlying these regulatory layers and examine evidence for their extensive functional interconnectivity. We discuss current advances in the understanding of the interplay between alternative splicing, chromatin remodeling, and cell cycle regulation in plants, highlighting how these processes might contribute to developmental plasticity and environmental adaptation while identifying key gaps that remain to be addressed.

## 1 Introduction

Plants are sessile organisms continuously exposed to environmental fluctuations that challenge their growth, development, and survival. Unlike animals, they cannot escape adverse conditions and therefore an important way they respond and adapt to the environment is by modulation of their growth and developmental plasticity (Schneider, 2022). This process involves the perception of endogenous and environmental cues, which trigger extensive transcriptional, post-transcriptional and epigenetic reprogramming, ultimately coordinating rates of cell division with cell expansion and differentiation (Qadir et al., 2026).

Cell cycle regulation functions not only as a mechanism controlling chromosome duplication and cell division, but also as an integrative system linking environmental signaling to growth and development. Under adverse environmental conditions, stress responsive signaling pathways frequently converge on the cell cycle machinery, reducing proliferative activity and redirecting metabolic resources from growth toward stress adaptation and survival (De Veylder et al., 2007; Qi and Zhang, 2020). Consequently, stress-induced modulation of the cell cycle, including transient arrest and endoreduplication, contributes directly to developmental, morphological, and physiological plasticity in plants (Carneiro et al., 2021; Sablowski and Gutierrez, 2022).

Beyond transcriptional regulation, plants also rely on post-transcriptional mechanisms to respond to fluctuating environmental conditions. Alternative splicing (AS) plays a fundamental role in plant adaptive responses to diverse abiotic stresses (Ganie and Reddy, 2021; Liu et al., 2022; Alhabsi et al., 2025). By generating multiple transcript isoforms from a single gene, AS greatly expands transcriptomic and proteomic diversity, contributing to functional plasticity in higher eukaryotes (Ganie and Reddy, 2021; Muhammad et al., 2023). In plants, approximately 70% of multi-exonic genes undergo AS, highlighting its importance as a regulatory mechanism controlling development and stress adaptation (Martín et al., 2021; Liu et al., 2022; Alhabsi et al., 2025). Under stress conditions, global shifts in splicing patterns frequently result in intron retention or transcripts with premature stop codons, reducing the production of pro-proliferative proteins and favoring cell cycle arrest (Muhammad et al., 2023).

Epigenetic regulation links environmental cues to gene expression and cell-fate decisions without altering the DNA sequence. In plants, DNA methylation, histone modifications, chromatin remodeling, and regulatory non-coding RNAs shape chromatin accessibility and transcription during development and stress responses (Dong et al., 2020; Godwin and Farrona, 2020; Vivek Hari Sundar et al., 2024). Among these mechanisms, histone modifications are key determinants of chromatin state. Active marks, such as histone H3 lysine 4 trimethylation (H3K4me3) and histone H3 lysine 36 trimethylation (H3K36me3), are generally associated with transcriptionally permissive chromatin, whereas repressive marks, including histone H3 lysine 9 dimethylation (H3K9me2) and histone H3 lysine 27 trimethylation (H3K27me3), are typically linked to heterochromatin formation and stable gene repression (Dong et al., 2020; Chujo and Scott, 2014; Dvořáčková et al., 2024).

In this context, stress-induced splicing changes do not occur independently of the transcriptional and chromatin environment. Because pre-mRNA splicing is often coupled to transcription, epigenetic mechanisms can influence gene accessibility, RNA polymerase II dynamics, and splice-site selection, thereby reshaping both transcriptional output and isoform profiles of genes involved in stress responses, genome stability, and cell proliferation (Naftelberg et al., 2015; Pajoro et al., 2017; Lucibelli et al., 2022; Mandadi et al., 2023). Chromatin dynamics are also tightly coordinated with plant cell cycle progression, including G1/S transcriptional control, DNA replication, G2/M-specific transcription, chromatin duplication, and mitotic chromosome organization (Sanchez et al., 2008; Desvoyes et al., 2014).

Despite substantial progress in dissecting cell cycle regulation, AS, chromatin-based control, and environmental stress responses, how these layers are integrated to shape plant growth decisions remains largely unknown. Here, we propose the AS–cell cycle axis as an emerging regulatory hub that links environmental perception, chromatin context, RNA processing, genome stability, and developmental plasticity. This framework provides a conceptual advance by explaining how stress-induced molecular reprogramming may be translated into adaptive changes in proliferation, endoreduplication, differentiation, and growth. By defining this interface, the review highlights a critical frontier in plant biology and outlines integrative approaches needed to move the field toward mechanistic and predictive models of plant adaptation to climate-related stresses. Such knowledge may also provide a foundation for future biotechnological strategies aimed at improving crop resilience.

## 2 Cell cycle regulation in plant growth and environmental responses

Plant growth and development are predominantly postembryonic processes sustained by stem cell populations in meristems, which divide and differentiate to generate new tissues and organs (Schwartz et al., 2021). These processes depend on the coordinated regulation of cell proliferation, expansion, and differentiation, which plants determine the spatial organization of tissues and organs in response to endogenous signals and environmental cues (Gonzalez et al., 2012; Sablowski and Gutierrez, 2022).

**Figure 1:**
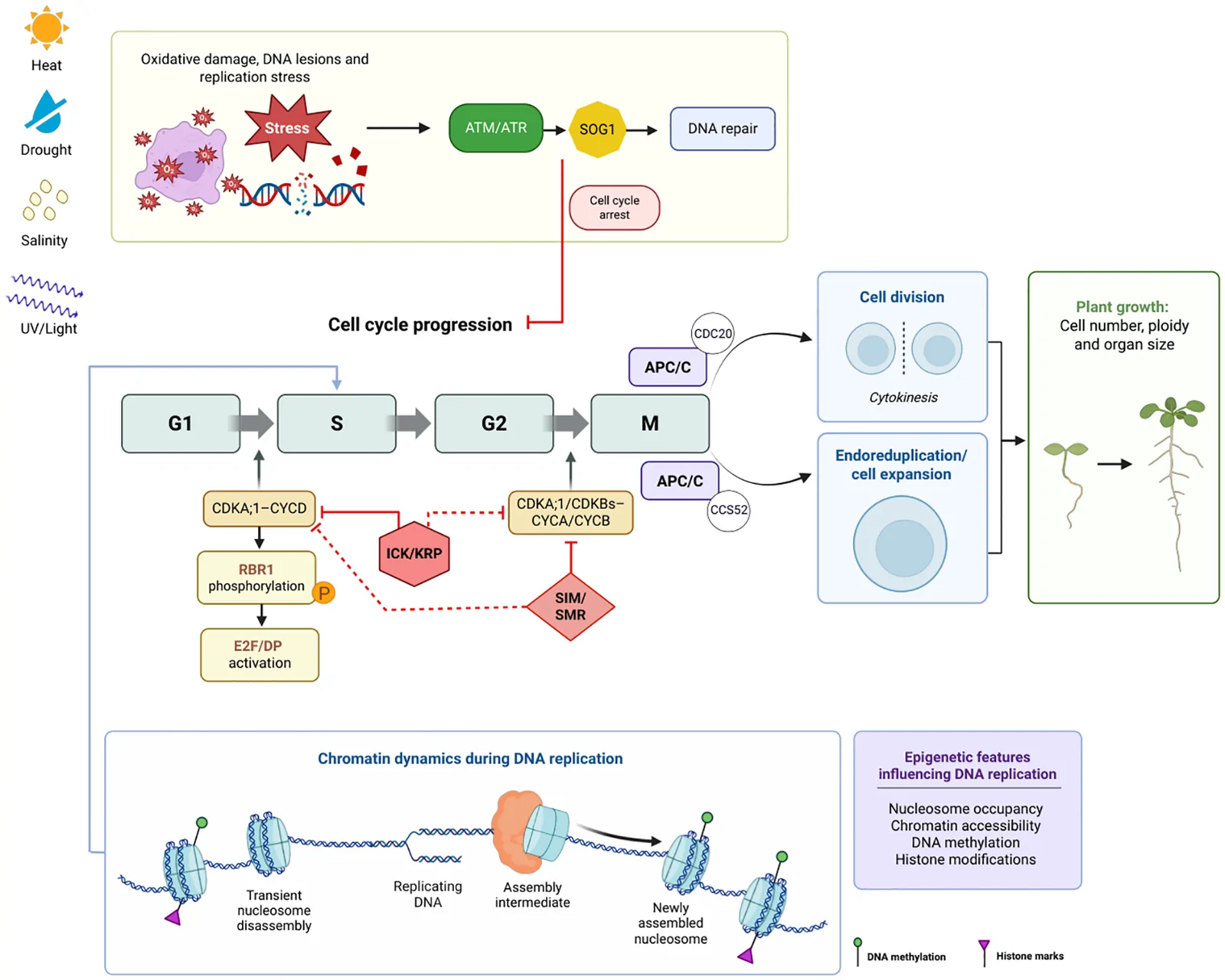
Regulation of plant cell cycle progression by environmental stresses and DNA replication dynamics. Plant cell cycle progression through the G1, S, G2, and M phases is controlled by cyclin-dependent kinase (CDK)–cyclin (CYC) complexes and their inhibitors. During the G1/S transition, CYCLIN-DEPENDENT KINASE A;1 (CDKA;1)–D-TYPE CYCLIN (CYCD) complexes phosphorylate RETINOBLASTOMA-RELATED 1 (RBR1), thereby relieving RBR1-mediated repression of E2 FACTOR/DIMERIZATION PARTNER (E2F/DP) transcription factors and promoting the expression of genes required for S-phase entry and DNA replication. Mitotic entry and progression are primarily driven by CDKA;1- and CYCLIN-DEPENDENT KINASE B (CDKB)-containing complexes associated with A- and B-type cyclins (CYCA and CYCB, respectively). INTERACTOR/INHIBITOR OF CYCLIN-DEPENDENT KINASE/KIP-RELATED PROTEIN (ICK/KRP) and SIAMESE/SIAMESE-RELATED (SIM/SMR) proteins inhibit CDK–CYC activity; solid inhibitory lines indicate their predominant regulatory effects, whereas dashed inhibitory lines indicate additional member- or context-dependent effects on the alternative cell cycle module. During mitosis, the ANAPHASE-PROMOTING COMPLEX/CYCLOSOME (APC/C), associated with CELL DIVISION CYCLE 20 (CDC20), promotes the degradation of mitotic regulators, chromosome segregation, mitotic exit, and cytokinesis. Association of APC/C with CELL CYCLE SWITCH 52 (CCS52) maintains low mitotic CDK activity and can redirect cells toward the endocycle, resulting in endoreduplication and cell expansion. During S phase, DNA replication is coordinated with transient nucleosome disassembly ahead of the replication machinery and nucleosome reassembly behind it through parental histone recycling and the deposition of newly synthesized histones. Nucleosome occupancy, chromatin accessibility, DNA methylation, and histone modifications influence replication-origin activity, replication timing, replication-fork progression, cell cycle gene expression, and the maintenance of epigenetic states. Environmental stresses, including heat, drought, salinity, and ultraviolet radiation, may induce oxidative damage, DNA lesions, and replication stress. These perturbations activate signaling mediated by ATAXIA TELANGIECTASIA MUTATED (ATM), ATM AND RAD3-RELATED (ATR), and SUPPRESSOR OF GAMMA RESPONSE 1 (SOG1), promoting DNA repair and restricting cell cycle progression. Together, cell division, endoreduplication, and chromatin- and stress-dependent regulation determine cell number, ploidy, cell expansion, and organ growth. Created in BioRender. Araújo-lopes, B. (2026) https://BioRender.com/apqnuj0.

The eukaryotic cell cycle comprises the G1, S, G2, and M phases, and cells may exit into a quiescent G0 state in response to differentiation cues, genotoxic stress, or growth limitation (Velappan et al., 2017; Soni and Bacete, 2023). Cell cycle progression is controlled by cyclin-dependent kinases (CDKs) and their associated cyclins (CYCs), whose stage-specific complexes coordinate DNA replication, chromosome segregation, and cell division. Entry into S phase depends on the conserved CDK–cyclin–RB/E2F module. In plants, CDKA;1–CYCD complexes phosphorylate RETINOBLASTOMA-RELATED 1 (RBR1), relieving its repression of E2F/DP transcription factors and promoting the expression of genes required for DNA replication (Inzé and De Veylder, 2006; De Veylder et al., 2007; Komaki and Sugimoto, 2012). In *Arabidopsis thaliana* (Arabidopsis), CDKs are classified into seven groups, from CDKA to CDKG, reflecting the functional diversification of this kinase family in plants (Vandepoele et al., 2002; Menges et al., 2005; De Veylder et al., 2007). CDKA and CDKB proteins are the principal regulators of canonical cell cycle progression. CDKA;1 functions throughout the mitotic cycle, whereas the largely plant-specific CDKBs accumulate mainly during G2 and M phases and, together with A- and B-type cyclins, promote mitotic entry and progression (Dewitte and Murray, 2003; Inzé and De Veylder, 2006; De Veylder et al., 2007; **Figure 1**).

CDK activity is restrained by INTERACTOR/INHIBITOR OF CYCLIN-DEPENDENT KINASE/KIP-RELATED PROTEINS (ICK/KRPs), which are functionally related to animal CIP/KIP proteins, and the plant-specific SIAMESE/SIAMESE-RELATED (SIM/SMR) proteins. These inhibitors can suppress cell cycle progression and favor entry into the endocycle (Inzé and De Veylder, 2006; De Veylder et al., 2007; Shimotohno et al., 2021). Cell cycle transitions are also controlled by ubiquitin-mediated proteolysis. The conserved ANAPHASE-PROMOTING COMPLEX/CYCLOSOME (APC/C), together with co-activators such as CDC20 and CCS52, promotes chromosome segregation, mitotic exit, and the degradation of mitotic regulators (Inzé and De Veylder, 2006; De Veylder et al., 2007; Komaki and Sugimoto, 2012; **Figure 1**). Reduced mitotic CDK activity and APC/C–CCS52-mediated proteolysis can redirect cells toward endoreduplication, in which DNA replication occurs without mitosis or cytokinesis, increasing ploidy and supporting cell expansion and differentiation (Inzé and De Veylder, 2006; De Veylder et al., 2007; Shimotohno et al., 2021).

Cell cycle progression also depends on chromatin-based regulation of cell cycle gene expression and on coordinated chromatin reorganization during DNA replication and chromosome segregation (Sanchez et al., 2008; Costas et al., 2011). During S phase, nucleosomes are transiently disassembled ahead of replication forks and reassembled behind them through parental histone recycling and deposition of newly synthesized histones, contributing to the maintenance of chromatin organization and epigenetic states in daughter cells (Sanchez et al., 2008; Iglesias and Cerdán, 2016; **Figure 1**). In turn, nucleosome occupancy, chromatin accessibility, DNA methylation, and histone modifications influence replication-origin activity, replication timing, and fork progression (Costas et al., 2011; Sequeira-Mendes and Gutierrez, 2015). Consequently, defects in chromatin assembly, remodeling, or epigenetic maintenance can perturb cell cycle gene expression and replication dynamics, generate replication stress, and compromise genome stability (**Figure 1**).

Environmental stresses such as heat, drought, salinity, and ultraviolet radiation can induce oxidative damage and DNA lesions, impairing replication-fork progression and compromising genome integrity (Carneiro et al., 2021; Shimotohno et al., 2021). These effects activate ATAXIA TELANGIECTASIA MUTATED (ATM)- and ATM AND RAD3-RELATED (ATR)-dependent checkpoints, which signal through SUPPRESSOR OF GAMMA RESPONSE 1 (SOG1) to reduce CDK activity, delay cell cycle progression, and promote DNA repair (De Schutter et al., 2007; Yoshiyama et al., 2013, 2014; Yoshiyama, 2015; Carneiro et al., 2021; Shimotohno et al., 2021). Together, cell cycle regulators and chromatin-based mechanisms integrate environmental stress with proliferation, endoreduplication, and growth, thereby contributing to developmental plasticity.

## 3 Alternative splicing as a dynamic regulatory mechanism in plants

Due to its role in regulating multiple environmental responses, gene expression regulation by AS has received increasing attention as a mechanism to promote adaptive responses in plants under stress conditions. Therefore, manipulation of AS holds potential for developing plants with improved agronomic traits, including stress tolerance (Ganie and Reddy, 2021). Here, we present a brief overview of splicing factors and their direct or indirect modulation in responses to abiotic stresses.

### 3.1 Core mechanisms regulating alternative splicing

**Figure 2:**
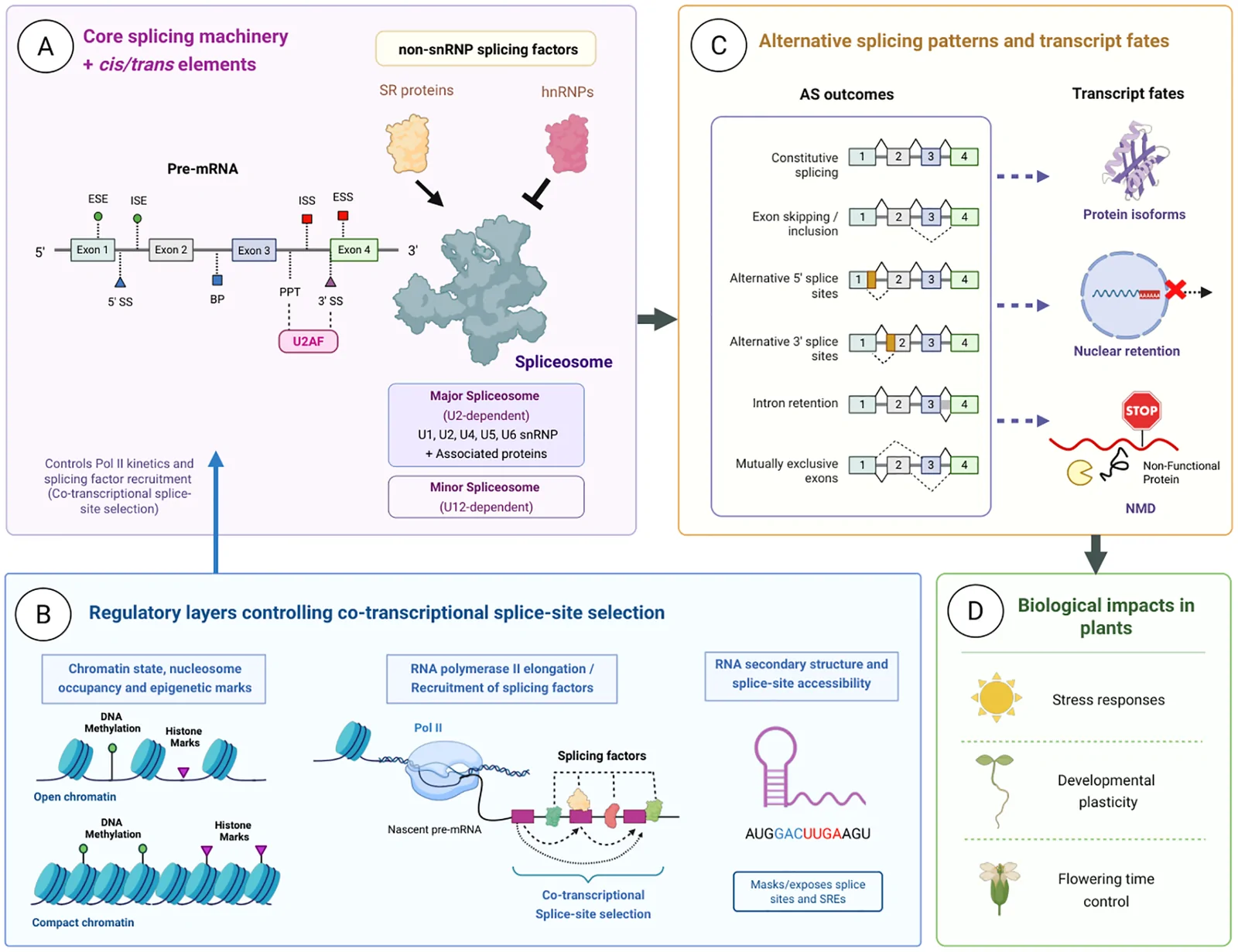
Core mechanisms and regulatory layers controlling alternative splicing (AS) in plants. **(A)** Core splicing machinery and *cis/trans* regulatory elements involved in splice-site recognition. Pre-mRNAs contain conserved *cis*-elements, including the 5′ splice site (5′SS), branch point (BP), polypyrimidine tract (PPT), 3′ splice site (3′SS), and splicing regulatory elements (SREs), such as exonic splicing enhancers (ESEs), intronic splicing enhancers (ISEs), exonic splicing silencers (ESSs), and intronic splicing silencers (ISSs). These *cis*-elements are recognized by *trans*-acting splicing factors. SR proteins generally bind ESEs and ISEs and promote splice-site recognition by recruiting or stabilizing spliceosomal components, thereby facilitating spliceosome assembly. In contrast, hnRNPs frequently bind ESSs and ISSs and repress splicing by masking splice sites, preventing the recruitment of spliceosomal factors, or antagonizing SR protein activity. These interactions, together with U2AF and the major U2-dependent and minor U12-dependent spliceosomes, regulate splice-site selection and transcript processing. **(B)** Regulatory layers modulating co-transcriptional splice-site selection. Chromatin state, nucleosome occupancy, DNA methylation, histone marks, RNA polymerase II elongation dynamics, recruitment of splicing factors, and RNA secondary structure influence splice-site accessibility and AS decisionsdecisions. **(C)** AS patterns and transcript fates. Differential splice-site usage generates multiple AS patterns, including exon skipping/inclusion, alternative 5′ and 3′ splice sites, intron retention, and mutually exclusive exons. These transcripts may encode distinct protein isoforms, be retained in the nucleus, be degraded through nonsense-mediated decay (NMD), or produce non-functional proteins. **(D)** Biological impacts of AS in plants, including stress responses, developmental plasticity, and flowering-time control. Created in BioRender. Araújo-lopes, B. (2026) https://BioRender.com/svc5env.

Pre-mRNA splicing is a fundamental post-transcriptional process in eukaryotes that removes non-coding introns and joins coding exons to generate mature messenger RNAs (mRNAs). This process is catalyzed by the spliceosome, a ribonucleoprotein complex composed of five small nuclear ribonucleoproteins (snRNPs; U1, U2, U4, U5, and U6), each containing a uridine-rich small nuclear RNA (snRNA) and associated proteins (Staiger and Brown, 2013; Alhabsi et al., 2025). During splicing, the spliceosome recognizes conserved *cis*-regulatory elements within the pre-mRNA, including the 5′ and 3′ splice sites, branch point sequences, polypyrimidine tracts, and splicing regulatory elements (SREs), such as enhancers and silencers (**Figure 2A**). In most introns, these core elements include conserved GU and AG dinucleotides at the 5′ and 3′ splice sites, respectively, a branch-point adenosine, and a polypyrimidine tract upstream of the 3′ splice site (**Figure 2A**). SREs may act as exonic or intronic splicing enhancers or silencers, commonly classified as exonic splicing enhancers (ESEs), intronic splicing enhancers (ISEs), exonic splicing silencers (ESSs), and intronic splicing silencers (ISSs) (**Figure 2A**). This recognition facilitates the catalysis of intron excision through sequential transesterification reactions (Muhammad et al., 2023; Alhabsi et al., 2025).

Splice-site recognition and exon inclusion are further modulated by non-snRNP splicing factors (SFs), particularly serine/arginine-rich (SR) proteins and heterogeneous nuclear ribonucleoproteins (hnRNPs), which bind regulatory RNA sequences to promote or repress spliceosome assembly (**Figure 2A**). Additional auxiliary factors, such as U2AF65 and U2AF35 (U2 auxiliary factor proteins), contribute to 3′ splice-site recognition by interacting with the polypyrimidine tract and the adjacent 3′ splice-site region (Wang and Brendel, 2006; Xiong et al., 2019; Muhammad et al., 2023). Mutations, insertions, or deletions within *cis*-regulatory elements can alter splice-site usage and reshape splicing patterns. This regulatory flexibility allows a single pre-mRNA to be processed in multiple ways, generating AS isoforms through exon skipping (ES), mutually exclusive exons (MXE), intron retention (IR), alternative 5′ donor splice sites (A5′SS), and alternative 3′ acceptor splice sites (A3′SS; **Figure 2C**). Notably, exon skipping predominates in animals, whereas intron retention is the most prevalent AS event in plants (Ner-Gaon et al., 2004; Barbazuk et al., 2008; Pan et al., 2008; Alhabsi et al., 2025). In addition to the major U2-dependent spliceosome, eukaryotes also possess a minor U12-dependent spliceosome that processes a small subset of non-canonical introns (Alhabsi et al., 2025).

Beyond sequence-dependent regulation, RNA secondary structure can influence AS by masking or exposing splice sites and regulatory motifs (**Figure 2B**). Although traditionally described as post-transcriptional, splicing is now recognized as largely co-transcriptional, with transcription and splicing being physically and functionally coupled in plants (Jabre et al., 2019; Muhammad et al., 2023). Splicing decisions are further modulated by chromatin organization and epigenetic modifications, including DNA methylation, nucleosome occupancy, histone modifications, and RNA polymerase II CTD-dependent recruitment of RNA-processing factors, which influence co-transcriptional splice-site selection by modulating RNA polymerase II elongation and recruiting splicing factors to chromatin (Godoy Herz and Kornblihtt, 2019; Jabre et al., 2019, 2022; Rigo et al., 2020; Liu et al., 2022; Jin et al., 2023; **Figure 2B**). Together, these regulatory layers establish AS as a highly dynamic process controlled by the coordinated action of RNA sequence features, splicing factors, RNA structure, and chromatin state. The resulting transcript isoforms may be translated into distinct protein variants, retained in the nucleus, or targeted for degradation through nonsense-mediated mRNA decay (NMD; **Figure 2C**) (Muhammad et al., 2023). Through these effects on transcript fate and protein output, AS contributes to key biological processes in plants, including stress responses, developmental plasticity, and flowering time control (**Figure 2D**). Consequently, AS expands transcriptomic diversity and contributes to the fine regulation of gene expression and proteome complexity in eukaryotic cells (Kornblihtt et al., 2013; Reddy et al., 2013). However, compared with animals, co-transcriptional splicing in plants remains less explored, particularly in developmental, tissue-specific, and condition-dependent contexts.

### 3.2 Environmental regulation of alternative splicing

By remodeling AS patterns, environmental fluctuations increase isoform diversity and position AS as a major regulatory mechanism underlying plant responses to abiotic stresses (Alhabsi et al., 2025; Liu et al., 2025). Genetic studies further demonstrate that mutations in spliceosome components frequently disrupt development and alter stress responsiveness, reinforcing the central role of splicing regulation in environmental adaptation (Liu et al., 2022; Guo et al., 2023).

A comprehensive overview of these components is presented in **Table 1**, which summarizes the main phenotypes and stress responses of spliceosome components and splicing regulators for which mutant-based evidence supports their involvement in stress regulation. Within this framework, core spliceosomal components and splicing regulators, particularly snRNP-associated factors, Sm core and Sm-like (LSM) proteins, tri-snRNP components, and SR proteins, emerge as consistent genetic determinants of stress responsiveness in plants.

**Table 1:** Spliceosome-associated factors and alternative splicing regulators involved in plant abiotic stress responses.

Functional class/complex	Splicing factor	Function	Mutant and associated phenotype	Related stress processes	Key references
Core spliceosomal component/snRNP	U1A	Component of the U1 snRNP complex; participates in pre-mRNA recognition and early spliceosome assembly.	*atu1a* mutants exhibit little or no evident developmental phenotype under standard conditions.	*atu1a* mutants exhibit hypersensitivity to salinity, reduced germination under NaCl, and reduced growth under salt stress.	Gu et al., 2018
Core spliceosomal component/U2 auxiliary factor	U2AF65A/B	U2 auxiliary factor; recognizes the polypyrimidine tract at the 3′ splice site and promotes U2 snRNP recruitment during early spliceosome assembly.	The *atu2af65a* mutant exhibits late flowering, reduced vegetative growth, decreased fertility, altered floral development, and splicing defects in several transcripts. The *atu2af65a atu2af65b* double mutant is inviable due to defects in male gametophyte development and impaired pollen tube growth.	*U2AF65A/B* mutants exhibit altered flowering responses under elevated temperature.	Park et al., 2019; Xiong et al., 2019
Core spliceosomal component/U4/U6 snRNP	RDM16/PRP3	Component of the U4/U6 snRNP complex; participates in the assembly and stability of the U4/U6.U5 tri-snRNP during catalytic spliceosome formation.	*rdm16–1* and *rdm16–4* mutants exhibit global splicing defects, smaller leaves, smaller siliques, wrinkled or rounded leaves, and developmental abnormalities.	Mutants exhibit hypersensitivity to salinity during germination, ABA hypersensitivity, and heat-stress sensitivity. They also show defective splicing of 18 of the 20 heat shock factor (HSF) genes.	Huang et al., 2013a; Ma et al., 2025
Core spliceosomal component/U4/U6.U5 tri-snRNP	STA1/PRP6	STA1 is the plant homolog of PRP6 and a component of the U4/U6.U5 tri-snRNP complex.	*sta1–1* mutants exhibit slower growth, reduced plant size, narrower leaves, delayed vegetative development, and late flowering.	Mutants show strong cold sensitivity and splicing defects in *COR15A*, required for low-temperature tolerance. Also required for splicing heat-responsive genes (HSFA3, HSA32, HSPs).	Lee et al., 2006; Kim et al., 2017; Yu et al., 2016
Core spliceosomal component/U4/U6 snRNP	PRP31	Component of the U4/U6 snRNP complex; participates in the formation and stability of the U4/U6.U5 tri-snRNP, which is essential for spliceosome activation.	*prp31* mutants exhibit global splicing defects, impaired growth, smaller rosette leaves, shorter petioles, reduced plant size, and developmental alterations.	Mutants exhibit increased sensitivity to cold, reduced germination, salt hypersensitivity, and ABA hypersensitivity.	Du et al., 2015
Core spliceosomal component/Sm proteins	SmE1/SmEb	Core component of the Sm ring of snRNPs; participates in the assembly and stability of snRNPs involved in pre-mRNA splicing.	*smeb-1* and *smeb-2* mutants exhibit reduced growth, lower survival, stronger developmental inhibition, intron retention, and incorrect splicing of several transcripts.	Mutants exhibit salt hypersensitivity and ABA hypersensitivity during germination.	Hong et al., 2021, 2023
Core spliceosomal component/LSm complex	LSM8	Component of the nuclear LSM2–8 complex; binds U6 snRNA, stabilizes the U6 snRNP, and participates in spliceosome assembly and activity, thereby regulating constitutive and alternative splicing.	*lsm8–1* and *lsm8–2* mutants exhibit intron retention and enrichment of mis-spliced transcripts related to stress and development.	Mutants exhibit reduced growth under NaCl conditions, reduced fresh mass compared with wild type, increased sensitivity to saline environments, and compromised growth and development under cold conditions.	Carrasco-López et al., 2017
Core spliceosomal component/LSm complex	LSM5/SAD1	Component of the nuclear LSm complex associated with U6 snRNA; contributes to spliceosome stability and function.	The hypomorphic *lsm5/sad1* mutant shows partial loss of function and demonstrates that LSM5 regulates proper RNA processing and stress-responsive gene expression under abiotic stress conditions.	Mutants exhibit hypersensitivity to salinity and ABA, together with alterations in RNA processing.	Cui et al., 2014; Okamoto et al., 2016
Core spliceosomal component/LSm complex	LSM4	LSM4 integrates the LSM2-8/U6 snRNP complex. They cooperate for spliceosome assembly and stability pre-mRNA processing.	The *lsm4* mutant exhibits early flowering compared with wild-type plants, consistent with a role for LSM4 in the regulation of flowering-related genes.	The mutant exhibits salt hypersensitivity, reduced growth, and stronger developmental inhibition under salt stress.	Zhang et al., 2011
SR splicing regulator	SR45	Serine/arginine-rich splicing factor that regulates alternative splicing by contributing to splice-site recognition, exon selection, and interactions with spliceosome components.	*sr45–1* mutants exhibit smaller plants, narrower leaves, shorter roots, late flowering, reduced fertility, fewer seeds, lower germination rates, and reduced cotyledon greening.	Mutants are more sensitive to salt and exhibit ABA hypersensitivity during germination and early growth, together with altered expression or splicing of ABA-responsive genes.	Carvalho et al., 2010; Albaqami et al., 2019
SR splicing regulator	SR45a	SR-family protein and non-snRNP splicing regulator involved in splice-site recognition, recruitment of spliceosome components, spliceosome assembly, and AS regulation.	The *sr45a* mutant does not show major morphological defects under standard conditions and has been described as a negative regulator of salt tolerance.	*sr45a* mutants exhibit higher germination rates on salt-containing media, increased cotyledon greening, and reduced sensitivity to salt stress, whereas SR45a overexpression decreases salt tolerance.	Li et al., 2021a
SR splicing regulator	RS33^1^	SR-like alternative splicing regulator involved in splice-site selection and recognition of pre-mRNA regulatory elements.	*rs33* mutants display a mild phenotype, with slight delay in germination and minor alterations in early growth, but no severe developmental defects under standard conditions.	Mutants exhibit hypersensitivity to salt and cold, reduced germination and seedling growth under stress, and altered splicing of numerous stress-responsive genes.	Butt et al., 2022
SR splicing regulator	SCL30a	SR splicing regulator that binds pre-mRNA regulatory elements and modulates splice-site selection.	*scl30a-1* and *scl30a-2* mutants exhibit smaller seeds, slower germination, and increased activation of genes associated with seed maturation and ABA responses.	Mutants exhibit hypersensitivity to ABA-induced germination inhibition and a marked reduction in germination under salt stress.	Laloum et al., 2023

This table summarizes core spliceosomal components and regulatory splicing factors with *Arabidopsis thaliana* mutant-based evidence linking them to developmental phenotypes and abiotic stress responsiveness.

^1^ Result obtained from a study conducted in rice (*Oryza sativa*).

Mutations affecting proteins associated with multiple spliceosomal subcomplexes, including Sm core proteins, the U4/U6.U5 tri-snRNP component PRP31 (pre-mRNA processing factor 31), and U6-associated LSM proteins such as LSM4, LSM5, and LSM8, frequently result in hypersensitivity to salinity, abscisic acid (ABA), cold, or freezing conditions (Zhang et al., 2011; Cui et al., 2014; Okamoto et al., 2016; Carrasco-López et al., 2017). These mutants commonly exhibit impaired germination, reduced growth, decreased survival under stress conditions, and widespread defects in pre-mRNA splicing, particularly affecting transcripts involved in stress signaling and adaptation (**Table 1**). Similarly, the Arabidopsis Sm core protein SmE1 plays a direct role in ABA-mediated stress responses, and its loss of function results in extensive intron retention and aberrant splicing of numerous transcripts involved in stress signaling and development (Hong et al., 2021; Hong et al., 2023).

SR proteins are involved in pre-mRNA splicing and other aspects of RNA metabolism (Graveley, 2000; Reddy, 2007). In plants, several members of this family are also functionally linked to abiotic stress signaling. Mutants in SR45, SR45a, and related SR proteins display altered sensitivity to salinity, cold, heat, or ABA, highlighting their role as modulators of stress-responsive AS and adaptive stress responses (Carvalho et al., 2010; Albaqami et al., 2019; Li et al., 2021a; Butt et al., 2022; Laloum et al., 2023).

Together, the genetic evidence obtained from these mutants demonstrates that plant stress adaptation depends not only on auxiliary splicing regulators but also on the integrity of the core spliceosomal machinery itself (Laloum et al., 2018; **Table 1**). The stress-sensitive phenotypes observed in mutants affecting Sm and LSm proteins, tri-snRNP components, and SR splicing factors indicate that multiple layers of the splicing machinery contribute to the coordinated regulation of gene expression under adverse environmental conditions. These defects are frequently associated with widespread alterations in pre-mRNA processing, including the mis-splicing of key stress-responsive transcripts, ultimately compromising plant growth, development, and survival. Thus, beyond its canonical role in RNA processing, the spliceosome acts as a dynamic regulatory hub that integrates environmental signals with developmental and physiological responses, thereby contributing to plant adaptive plasticity under stress conditions (**Table 1**). Because plant adaptation to environmental stress is closely linked to adjustments in growth and proliferative activity, the widespread involvement of splicing factors in stress responses also suggests a potential connection with cell cycle regulation. Environmental cues such as salinity, drought, temperature fluctuations, and ABA signaling are well-established modulators of meristem activity and cell proliferation, often triggering transient cell cycle arrest and extensive transcriptional reprogramming (Qi and Zhang, 2020; de Luxán-Hernández et al., 2022; Huang et al., 2026). In this context, AS may represent an additional regulatory layer linking stress adaptation to growth control and cell cycle progression.

## 4 Alternative splicing as a regulator of cell cycle dynamics

Increasing evidence indicates that splicing regulation and cell cycle control are tightly interconnected, forming a bidirectional network in which splicing factors and AS events modulate cell cycle transitions, while cell cycle dynamics influence splicing outcomes. Although this relationship has been extensively investigated in animal systems, particularly in mammals, its underlying mechanisms remain comparatively less explored in plants. In this section, we discuss this relationship across eukaryotes, highlighting current knowledge, emerging evidence in plants, and key mechanisms that remain poorly understood.

### 4.1 Alternative splicing and cell cycle control as interconnected regulatory processes in eukaryotes

Because nearly all expressed genes undergo pre-mRNA splicing, cell cycle progression is strongly dependent on spliceosome integrity and RNA-processing efficiency. In animal systems, partial reduction of spliceosome activity preferentially affects later stages of the cell cycle, including G2 and mitosis, whereas stronger depletion can induce earlier arrest, indicating a dosage-dependent requirement for spliceosome function throughout cell cycle progression (Karamysheva et al., 2015). More broadly, AS has emerged as a major regulatory layer controlling cell identity, developmental transitions, and proliferative programs across eukaryotes (David and Manley, 2010; Kalsotra and Cooper, 2011).

Beyond this general requirement, transcriptome-wide studies have shown that AS is dynamically and periodically regulated during cell cycle progression, providing an additional regulatory layer that can operate partly independently of transcriptional oscillations (Dominguez et al., 2016a). In human cells, several RNA-processing factors also function as direct regulators of proliferation, checkpoint signaling, genome stability, and mitotic progression. These include spliceosomal components, SR proteins, hnRNPs, splicing-associated kinases, and regulatory factors that control the splicing or expression of key cell cycle genes, including cyclins, CDKs, checkpoint regulators, and mitotic factors (David and Manley, 2010; Zhou and Fu, 2013).

Mechanistically, the AS–cell cycle interface can be organized into three major regulatory axes. First, spliceosome activity itself is remodeled across cell cycle phases, particularly during mitosis, when global splicing inhibition and changes in spliceosome assembly occur (Shin and Manley, 2002; Dominguez et al., 2016b). Second, cell cycle kinases and checkpoint pathways regulate splicing factors and spliceosomal components through phosphorylation, thereby coupling RNA processing to cell cycle stage and DNA damage responses (Colwill et al., 1996; Choi et al., 2014; Dominguez et al., 2016b; Hluchý et al., 2022). Third, AS reciprocally regulates cell cycle progression by controlling isoform selection of genes involved in G1/S transition, DNA replication, G2/M progression, mitosis, cytokinesis, and genome stability (Kurokawa et al., 2014; Mao et al., 2019; Xu et al., 2023).

Accordingly, **Table 2** summarizes literature-supported factors with experimentally validated roles in both RNA splicing and cell cycle progression. The STRING-based network presented in **Box 1** provides a complementary interaction framework, but the table emphasizes functional evidence rather than network membership alone. Together, these resources provide an overview of the molecular factors and regulatory mechanisms currently known to connect AS, RNA processing, and cell cycle control in eukaryotic systems.

**Table 2:** Dual-function regulators of alternative AS and cell cycle progression in eukaryotes.

Factor	ID (Ensembl)	Type	Role in splicing	Role in cell cycle	Functional Evidence	Observed phenotype	Cell cycle phase affected	Organism	Key references
CDC5L	ENSG00000096401	Cell division cycle 5 like	Part of PRP19/CDC5L complex, essential for the catalytic step II of pre-mRNA splicing. Involved in pre-mRNA splicing function in chondrogenic genes and Wee1.	Regulates S-phase progression and G2/M checkpoint signaling through ATR-dependent pathways and control of CDK1/cyclin B1 accumulation.	Western Blot; Immunohistochemistry (IHC); Fluorescence *In Situ* Hybridization (FISH); TUNEL, Flow Cytometry; CCK-8	Controls cell growth, proliferation, cell cycle checkpoints, promotes chondrogenesis and cartilage growth. Low levels of CDC5L lead to cell cycle arrest, incorrect spindle assembly, impaired kinetochore-microtubule attachment, chromosome misalignments and DNA damage.	S, G2/M phases	*Homo sapiens*	Boudrez et al., 2000; Grote et al., 2010; Zhang et al., 2009; Mu et al., 2014; Jokoji et al., 2021
USP39	ENSG00000168883	Ubiquitin specific peptidase 39	Controls pre-mRNA splicing of Aurora B. USP39 regulates splicing of several genes (like FoxM1, PLK1 and cyclin B1 interactor), including autophagy-related genes. Stabilizes key regulatory proteins.	Deubiquitinates and phosphorylation Cyclin B1. Stabilizes β-catenin and suppresses the pre-mRNA maturation of E3 ligase TRIM26. Regulates DNA damage. Stabilizes CHK2 and modulates p53/p21/CDC2/cyclin B1 promoting proliferation.	Immunofluorescence; Immunoblotting; RNA pull-down; *Flow cytometry*; Northern Blot; Western Blot; Immunoprecipitation; CCK-8; Immunohistochemistry	Normal expression levels lead to cell proliferation and growth. Low levels of USP39 lead to inhibited growth and G2/M arrest.	G2/M	*Homo sapiens; Mus musculus*	Yuan et al., 2015; Li et al., 2021c; Zhang et al., 2022; Cui et al., 2023; Yuan et al., 2025
SYF2	ENSG00000117614	SYF2 pre-mRNA splicing factor	Controls the splicing program of Epithelial cell transforming 2 (ECT2), leading to drug resistance associated with higher levels of ECT2-Ex5+ isoform. Required for astrocyte activation and neuronal apoptosis by mediating expression of cyclin D1. SYF2 modulates transcriptional and post-transcriptional controls of α-tubulin.	Cyclin D1 interactor. Regulates expression of α-tubulin, participate in the phosphorilation of Chk1. Total loss of function for SYF2 (p29) resulted in embryonic lethality, aberrant cell cycle checkpoint response and the mRNA levels of α-tubulin and Chk1 were reduced. Induces the expression of PCNA and cyclin D1 and modulates cell proliferation.	Biochemical assay; RNA Immunoprecipitation (RIP); Mass Spectrometry; Flow Cytometry; Western Blot; Immunocytochemistry; Immunohistochemistry; CCK-8	Involved in the expression of α-tubulin, cyclin D1, Chk1, and PCNA. When absent, leads to impaired cell growth and cell cycle arrest at G1/S, G0/G1 and G2/M phases. Total loss of function is embryonic lethal and leads to aberrant cell cycle checkpoint response. Knockdown leads to premature chromatin condensation and apoptosis.	G0/G1, G1/S and G2/M phases	*Homo sapiens; Mus musculus; Saccharomyces cerevisiae*	Chu et al., 2009; Chen et al., 2012; Xu et al., 2014; Guo et al., 2014; Zhou et al., 2014; Zhang et al., 2015; Shi et al., 2017; Tanaka et al., 2020
PHF5A	ENSG00000100410	Plant homeodomain (PHD)–finger domain protein 5A	Core component of the SF3b complex; recognizes the branch-point sequence during pre-mRNA splicing and regulates alternative splicing.	Regulates G1/S cell cycle progression through alternative splicing of cell cycle regulators such as SKP2, CHEK2 and ATR. PHF5A depletion induces G0/G1 arrest and reduces proliferation.	siRNA-mediated knockdown in lung adenocarcinoma cells reduced proliferation and altered splicing of cell cycle genes; flow cytometry showed G0/G1 arrest.	Reduced proliferation, clonogenicity, migration and invasion; induction of G0/G1 cell cycle arrest and increased cisplatin-induced apoptosis after PHF5A silencing.	G0/G1 transition (G1/S checkpoint)	*Homo sapiens*	Mao et al., 2019; Teng et al., 2017; Strikoudis et al., 2016
PUF60	ENSG00000179950	Poly(U)-binding splicing factor 60	Promotes exon inclusion by binding U-rich intronic regions adjacent to regulated exons; regulates skipped-exon events in genes associated with mitotic progression, especially promoting inclusion of exon 3 in CDC25C pre-mRNA.	Regulates G2/M transition through alternative splicing of mitotic cell cycle genes, particularly CDC25C.	siRNA/CRISPR depletion, RNA-seq splicing analysis, RT-PCR validation, minigene assays, CLIP-PCR, flow cytometry, rescue experiments, and mouse tumor models.	Reduced proliferation, decreased clonogenicity, G2/M arrest, increased apoptosis, and impaired tumor growth after PUF60 depletion.	G2/M transition	*Homo sapiens*	Xu et al., 2023
SRSF3	ENSG00000112081	Serine/Arginine-Rich Splicing Factor 3	Regulates alternative splicing of cancer/cell cycle-related transcripts (HIPK2, ILF3, ETV1 and NDE1); generally acts through binding to splicing enhancer/silencer elements to control exon inclusion/skipping.	Supports G1/S progression by maintaining expression of G1/S regulators (cyclin D1, cyclin D3, cyclin E1, E2F1 and E2F7); contributes to tumor-cell proliferation and survival.	siRNA/shRNA knockdown, flow cytometry, BrdU incorporation, microarray/RNA-seq splicing analysis, RT-PCR/qPCR, western blot, apoptosis assays, CRISPR/Cas9 isoform editing, transformation assays and xenograft/tumor models.	SRSF3 depletion causes G1 arrest, reduced S-phase entry, reduced proliferation, apoptosis, impaired self-renewal/tumorigenicity, and altered oncogenic splice isoforms.	G1/S transition	*Homo sapiens*	Kurokawa et al., 2014; Jia et al., 2010, 2019; Song et al., 2019; He and Zhang, 2015
CDC40 (PRP17)	ENSG00000168438	Cell division cycle 40	Second-step spliceosomal factor required for efficient splicing of specific cell cycle-related transcripts (ANC1, TUB1/TUB3 and CDCA5). Its depletion induces intron retention and accumulation of unspliced pre-mRNAs.	Regulates G1/S and G2/M progression through efficient splicing of cell cycle-associated transcripts involved in DNA replication, spindle assembly and mitotic progression, including ANC1, TUB1/TUB3 and CDCA5.	Null mutants, arrest-release assays, RT-PCR and *in vitro* splicing assays, FACS analysis, intron-removal rescue experiments, RNA-seq/transcriptome-wide splicing analysis, shRNA knockdown, and proliferation/apoptosis assays.	Yeast mutants exhibit G1/S delay, G2/M arrest and spindle defects, whereas CDC40-depleted lung cancer cells show intron retention, reduced proliferation and apoptosis.	G1/S and G2/M transitions	*Saccharomyces cerevisiae; Homo sapiens*	Ben-Yehuda et al., 2000; Chawla et al., 2003; Dahan and Kupiec, 2004; Kaplan and Kupiec, 2007; Hu et al., 2025
IK	ENSG00000113141	RED protein	Part of the spliceosome, involved in pre-mRNA splicing.	Needed for mitotic cell cycle progression. Required for spindle assembly checkpoint, through the recruitment of MAD1L1 and MAD2L1.	Pull-down assay; Mass spectrometry; Cryo-EM	Lack of IK leads to shorter mitotic timing, defects in SAC	Spindle Assembly Checkpoint	*Homo sapiens*	Yeh et al., 2012; Bertram et al., 2017
CLK1	ENSG00000013441	CDC-like kinase 1	Phosphorylates SR splicing factors (e.g., SRSF1/ASF-SF2) and regulates SR-dependent spliceosome activity, exon inclusion and intron removal during alternative splicing.	Coordinates periodic alternative splicing programs required for G1/S transition, late S/G2 progression, mitosis and cytokinesis, particularly through regulation of genes involved in DNA replication, checkpoint control and chromosome segregation.	Yeast two-hybrid assays, ASF/SF2 phosphorylation assays, TG003 inhibition, RNA-seq of synchronized cells, RT-PCR validation, shRNA knockdown, live-cell imaging, colony formation assays, and FACS analysis.	CLK1 inhibition/depletion causes intron retention, exon skipping, G1/S arrest, mitotic defects, impaired proliferation and cell death; CLK1 overexpression is associated with cancer progression.	G1/S transition, late S/G2 progression, mitosis and cytokinesis.	*Homo sapiens*	Colwill et al., 1996; Dominguez et al., 2016b
CDK11 (CDK11A/CDK11B)	ENSG00000008128/ENSG00000248333	Cyclin-dependent kinase; splicing-associated CDK	Phosphorylates SF3B1 during spliceosome activation, promoting transition from B complex to catalytically active Bact complex; CDK11 inhibition causes widespread intron retention and impaired pre-mRNA splicing.	Coordinates cell cycle progression through spliceosome activation and regulation of replication-dependent histone gene expression.	OTS964 inhibition, CDK11-resistant mutant rescue, RNA-seq/4SU-seq, RT-qPCR, SF3B1 phosphorylation assays, spliceosome activation analysis, co-IP, kinase assays, proliferation assays, and FACS.	Global intron retention, defective spliceosome activation, reduced proliferation, G2/M accumulation, and defective replication-dependent histone gene expression after CDK11 inhibition/depletion.	G2/M accumulation; S-phase-linked histone gene expression	*Homo sapiens*	Hu et al., 2003; Choi et al., 2014; Hluchý et al., 2022
HNRNPU	ENSG00000153187	Heterogeneous nuclear ribonucleoprotein U	Required for pre-mRNA splicing of several genes and RNA pol II elongation.	Controls transcrition and levels of CDK2, acting in mitotic progress. Interacts with DDX5 for transcriptional regulation.	Western Blot; CCK-8; Immunohistochemistry; ChIP	Knockout mutants exhibited misregulated gene expression and abnormal pre-mRNA splicing, chromosome misalignments, cell cycle arrest	G1/S phase	*Homo sapiens; Mus musculus*	Kim and Nikodem, 1999; Ma et al., 2011; Douglas et al., 2015; Ye et al., 2015; Nozawa et al., 2017; Liang et al., 2021
TXNL4A	ENSG00000141759	Thioredoxin-like protein 4A	Responsible for the splicing of several genes relevant to craniofacial and embryonic development. Low levels of TXNL4A expression leads to mis-splicing of TCF7L2 exon 4 and other mis-spliced genes.	Haploinsufficiency of TXNL4A was linked to defects in cranial neural crest cell formation in *Xenopus*. Single-cell RNA analysis identified TXNL4A as a potential biomarker for HCC.	Immunostaining; Karyotyping; Proliferation Assay; Cell migration; TUNEL; *In Situ* Hybridization	Core component of the U5 Splicing complex. Loss of function or haploinsufficiency leads to malformation and several human syndromes.	Not reported	*Homo sapiens; Xenopus*	Liu et al., 2006; Beauchamp et al., 2020; Wood et al., 2022; Park et al., 2022; Li et al., 2023
PRPF40A	ENSG00000196504	Pre-mRNA-processing factor 40 homolog A	Acts as an activator of cassette exon inclusion of splicing events with functional relevancy. Coregulates microexon splicing in mouse.	Co-expressed genes of the AMPK pathway were identified in GO and pathway analysis	Immunohistochemistry; CCK-8; Western Blot	Knockdown lead to inhibted cell proliferation and increased cell death.	Not reported	*Homo sapiens; Mus musculus*	Huo et al., 2019; Tan et al., 2024; Choudary and Norris, 2024
ZNF830	ENSG00000198783	Zinc finger protein 830	Possible role in pre-mRNA splicing, interacts with the XABS2 splicing complex.	Regulator of cell cycle and maintenance of genome integrity. Mitotic progression and cell survival.	Immunohistochemistry; ChIP; EMSA; Immunofluorescence	DNA damage repair in double-strand overhang	M phase	*Homo sapiens*	Kuraoka et al., 2008; Chen et al., 2018; Ahmad et al., 2025

The table summarizes proteins with experimentally supported roles in RNA splicing or spliceosome-associated regulation that also affect cell cycle progression, checkpoint signaling, proliferation, genome stability, or cell survival. The STRING-based network shown in **Box 1** was used as a complementary interaction framework, whereas factor inclusion was primarily guided by functional evidence from the literature.

Box 1 **The Alternative Splicing–Cell Cycle Interface in Plants: A Major Experimental Knowledge Gap.**

Although the functional interplay between alternative splicing (AS) and cell cycle regulation has been increasingly recognized in yeast and mammalian systems, this interface remains largely unexplored in plants. This gap is particularly relevant because plant developmental plasticity depends on the coordinated regulation of RNA processing, proliferative activity, genome stability, and environmental responses. To assess the current experimental support for AS–cell cycle connectivity and identify candidate regulatory hubs, we performed a comparative *in silico* interaction analysis integrating functional annotation and experimentally validated protein–protein interaction datasets from *Homo sapiens* and *Arabidopsis thaliana*.

Cell cycle-associated proteins were retrieved from QuickGO using the Gene Ontology term “cell cycle” (GO:0007049; Binns et al., 2009), whereas spliceosome-associated proteins were obtained from the Spliceosome Database (Cvitkovic and Jurica, 2013). Protein–protein interaction networks were then constructed in STRING (Szklarczyk et al., 2025), considering only experimentally supported physical interactions. To increase the robustness of the analysis, a high-confidence interaction score cutoff (≥0.9) was applied for *H. sapiens*. For Arabidopsis, a medium-high confidence threshold (≥0.7) was adopted to account for the comparatively limited availability of experimentally validated plant interaction data.

The human network revealed extensive connectivity between AS- and cell cycle -associated proteins, identifying 183 AS-related proteins interacting with 59 cell cycle-associated proteins. Among these, eight proteins displayed experimentally supported annotations in both splicing and cell cycle regulation, suggesting the presence of regulatory hubs potentially coordinating transcript processing with cell cycle progression. In contrast, the Arabidopsis network contained only 14 AS-associated proteins and three cell cycle-associated proteins that fulfilled the same selection criteria, with only one protein showing experimentally supported functions in both pathways. This striking disparity is unlikely to reflect a true biological absence of AS–cell cycle coordination in plants. Rather, it exposes the underrepresentation of plant experimental datasets, particularly those addressing physical interactions among spliceosome-associated proteins, cell cycle regulators, and stress-responsive signaling components.

This imbalance is especially important because several plant processes controlled by environmental cues, including meristem activity, DNA damage responses, replication stress, endoreduplication, and developmental reprogramming, require coordinated regulation of RNA processing and cell cycle progression. Thus, the limited number of experimentally validated AS–cell cycle interactions currently available for Arabidopsis should be interpreted not as evidence of biological simplicity, but as a critical research gap.

The proteins identified through this analysis provide a useful framework for prioritizing candidate regulators and highlight the need for integrative approaches combining transcriptomics, proteomics, interactomics, and functional genetics. Such strategies will be essential to uncover how AS contributes to cell cycle regulation, genome stability, and environmental adaptation in plants.

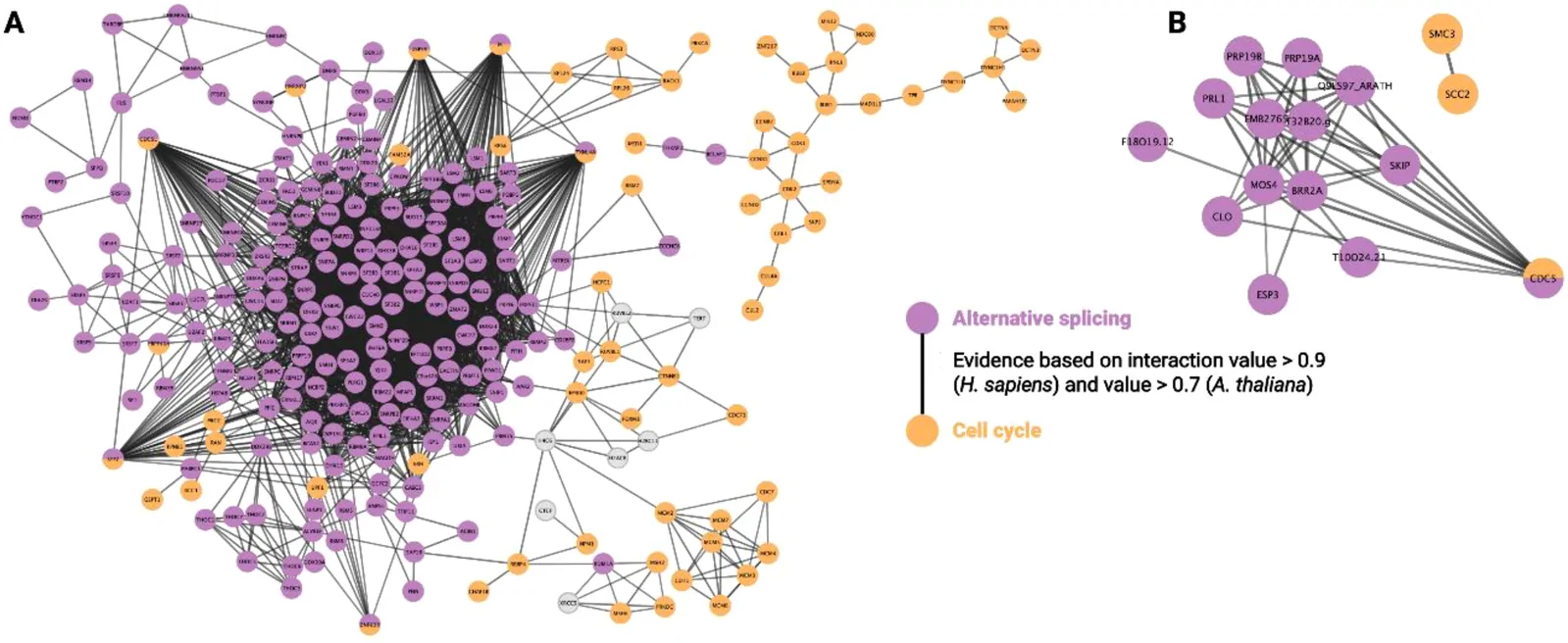

**Box 1 Figure** - Comparative interaction networks between alternative splicing (AS) and cell cycle components in *Homo sapiens* **(A)** and *Arabidopsis thaliana* **(B)**. Nodes represent proteins and edges indicate experimentally validated physical interactions. Purple, orange, and gray nodes correspond to AS-related proteins, cell cycle-associated proteins, and additional interacting partners, respectively.

Taken together, the AS–cell cycle interface is particularly well documented in animal and human systems, especially in cancer, where dysregulated splicing factors and altered AS programs frequently reshape pathways controlling proliferation, cell cycle progression, survival, migration, and therapy resistance (Mu et al., 2014; Oltean and Bates, 2014; Tsai et al., 2015; Dominguez et al., 2016b; Petasny et al., 2021). The factors summarized in **Table 2** converge on recurrent mechanisms, including spliceosome remodeling, phosphorylation-dependent regulation of splicing factors and spliceosomal components, and AS-mediated control of transcripts encoding checkpoint, proliferative, and mitotic regulators (Colwill et al., 1996; Shin and Manley, 2002; Choi et al., 2014; Dominguez et al., 2016b; Hluchý et al., 2022).

At the mechanistic level, cell division cycle 5-Like (CDC5L), a core component of the pre-mRNA Processing Factor 19 Complex (PRP19C; also known as the NineTeen Complex - NTC), and USP39 illustrate how spliceosome integrity contributes to G2/M progression, mitotic checkpoint control, and proliferative growth, whereas HNRNPU/SAF-A, CLK1, and CDK11 exemplify how transcriptional regulation, chromatin organization, kinase signaling, and spliceosome activity intersect during cell cycle progression (Van Leuken et al., 2008; Grote et al., 2010; Mu et al., 2014; Douglas et al., 2015; Yuan et al., 2015, 2025; Jokoji et al., 2021). Conversely, factors such as PHD finger protein 5A (PHF5A), poly(U)-binding splicing factor 60 (PUF60), serine/arginine-rich splicing factor 3 (SRSF3), and SYF2 pre-mRNA splicing factor (SYF2) show that AS can directly influence cell cycle control by regulating isoform production of transcripts involved in G1/S transition, DNA replication, G2/M progression, mitosis, drug resistance, and genome stability (Kurokawa et al., 2014; Mao et al., 2019; Tanaka et al., 2020; Xu et al., 2023). Thus, evidence from yeast and animal systems, including human cells, supports a robust bidirectional model in which spliceosome function, AS regulation, checkpoint signaling, and kinase networks are dynamically integrated to coordinate cell cycle progression and genome maintenance.

### 4.2 Molecular links between alternative splicing, cell cycle regulation, and developmental growth in plants

Defects in spliceosome components and splicing-associated regulators are frequently associated with impaired growth, developmental abnormalities, and changes in cell proliferation, cell expansion, or endoreduplication (Ali et al., 2007; Lin et al., 2007; Pei et al., 2007; Xu et al., 2012; Jiang et al., 2022; Liu et al., 2025). However, in plants, direct mechanistic evidence linking splicing dysfunction to specific cell cycle phases remains limited. Current studies support a broader, likely bidirectional relationship in which transcriptional activity and RNA-processing dynamics shape AS outcomes, while splicing-associated proteins can affect the expression or processing of genes involved in proliferation, endoreduplication, developmental transitions, and growth control (Lin et al., 2007; Kitsios et al., 2008; Zhao et al., 2017; Zhang et al., 2020; Jiang et al., 2022; Guo et al., 2024). Thus, AS and RNA processing may contribute to cell cycle associated developmental regulation in plants, although this interface remains less characterized than in animals.

**Figure 3:**
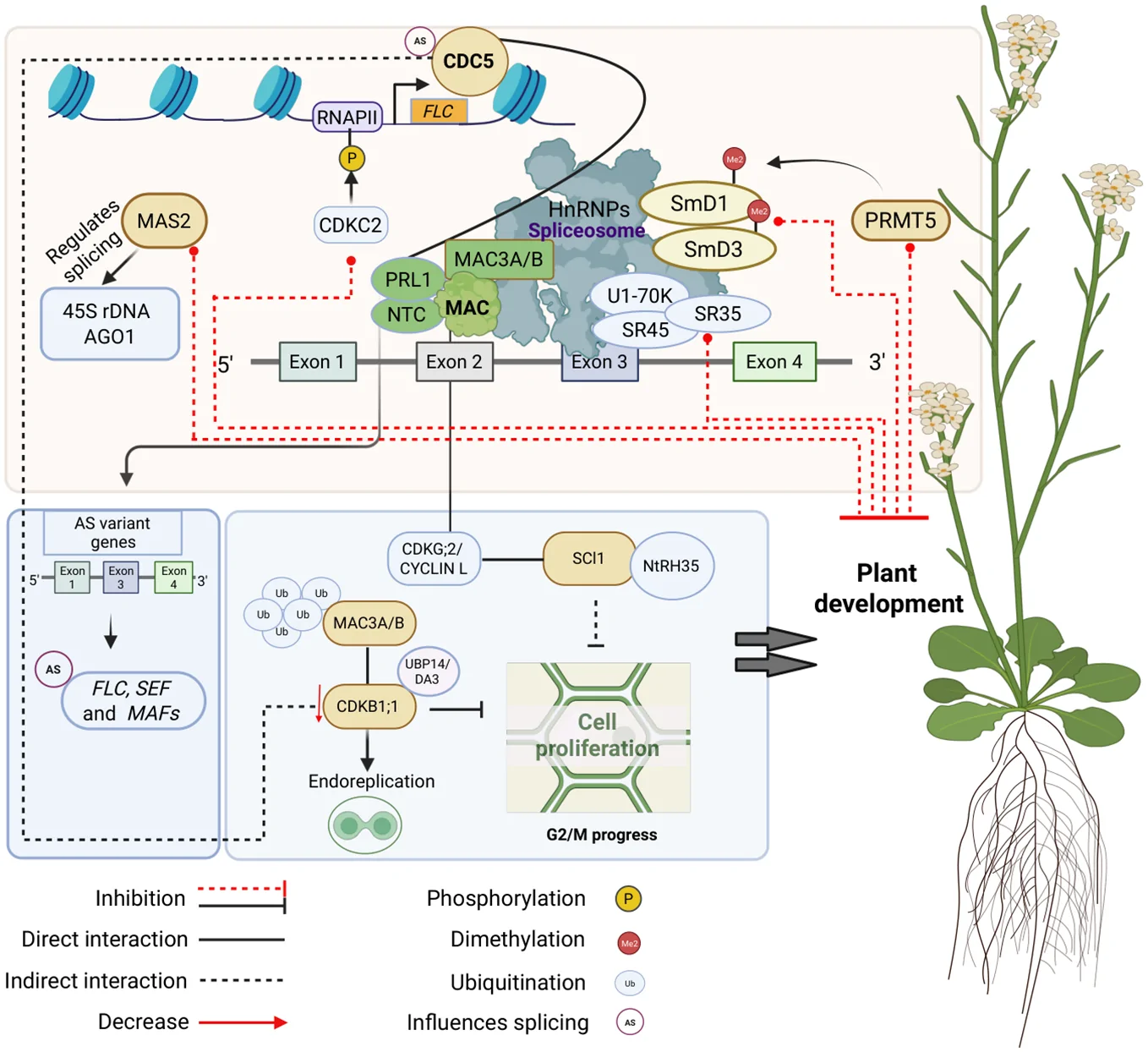
Spliceosome-associated factors coordinate cell cycle progression and development in plants. The diagram summarizes the regulatory network connecting pre-mRNA splicing, and cell proliferation in plants, with emphasis on *Arabidopsis thaliana* and selected examples from *Nicotiana tabacum*. Cell Division Cycle 5(CDC5) acts as a multifunctional factor associated with RNA polymerase II, transcriptional regulation, and spliceosome activity, influencing the expression and AS of developmental genes, including *FLOWERING LOCUS C (FLC), SERRATED LEAVES AND EARLY FLOWERING (SEF)*, and *MADS AFFECTING FLOWERING* genes (*MAF*). CDC5 also interacts with core spliceosomal components and the MOS4-associated complex (MAC), contributing to spliceosome assembly and pre-mRNA processing. Loss of CDC5 function is associated with reduced Cyclin-Dependent Kinase B1;1 (CDKB1;1) expression, impaired G2/M progression, and increased endoreduplication. METHYLASE ASSOCIATED SILENCING 2 (MAS2) contributes to epigenetic regulation by maintaining 45S rDNA silencing and nucleolar organization, supporting proper plant growth and development. In parallel, Protein Arginine Methyltransferase 5 (PRMT5/SKB1) mediates symmetric arginine dimethylation of Sm proteins and additional splicing factors, thereby modulating spliceosome function and AS outcomes. Several spliceosomal proteins, including MAC3A/B, Sm proteins D1 and D3 (SmD1 and SmD3), serine/arginine-rich splicing factors 45 and 35 (SR45 and SR35), U1 small nuclear ribonucleoprotein 70 kDa (U1-70K), and Pleiotropic Regulatory Locus 1 (PRL1), have been implicated in f developmental regulation, with individual factors affecting cell cycle progression, endoreduplication, and meristem activity. AS dependent regulation of cell cyclerelated genes, such as Cyclin-Dependent Kinase G2 (CDKG2), Cyclin L, Cyclin-Dependent Kinase B1 (CDKB1), Stigma/Style Cell-Cycle Inhibitor 1 (SCI1), and *Nicotiana tabacum* RNA Helicase 35 (NtRH35), contributes to the coordination of G2/M progression and cellular proliferation. Beyond its role in pre-mRNA splicing, MAC3A/B also functions as an E3 ubiquitin ligase that ubiquitinates by UBIQUITIN-SPECIFIC PROTEASE 14 (UBP14/DA3), thereby modulating the UBP14/DA3–CDKB1;1 pathway, which regulates endoreduplication and cell expansion. Collectively, these interconnected pathways integrate chromatin-associated regulation, RNA processing, and cell cycle control to modulate plant growth and development. Created in BioRender. Araújo-lopes, B. (2026) https://BioRender.com/7mlxg1a.

Based on experimentally supported studies, **Table 3** summarizes plant proteins implicated in AS, pre-mRNA processing, cell cycle associated processes, and developmental growth. Among these candidates, CDC5, MOS4-associated complex 3A/B (MAC3A/B), CDKC;2, and CDKG;2 provide the strongest links with cell cycle, including G2/M transition, endoreduplication, altered ploidy, cell division, and organ size (**Figure 3**). Arginine methyltransferase 5 (PRMT5) strongly connects spliceosome regulation with splicing fidelity and developmental robustness, whereas stigma/style cell-cycle inhibitor 1 (SCI1) links tissue-specific cell proliferation with spliceosome-associated protein networks. Other factors, including SR45, SCL proteins, the small nuclear ribonucleoproteins SmD1b and SmD3, U1-70K, METHYLASE ASSOCIATED SILENCING 2 (MAS2/NKAP), and CYCL/MOS12, are mainly supported as splicing or RNA-processing regulators whose loss affects growth, organogenesis, flowering, fertility, or embryo development, but without evidence for direct cell cycle phase control. Rather than defining a single linear pathway, these factors reveal a multilayered regulatory landscape in which spliceosome activity, AS control, transcription coupled RNA processing, and developmental cell cycle programs converge. In the following sections, we discuss these candidates individually to highlight how their integrated functions support an emerging framework linking AS and RNA processing to cell cycle-associated developmental regulation in plants.

**Table 3:** Plant splicing-associated factors linked to cell cycle-related growth and development.

Factor	ID (TAIR)	Type	Role in splicing	Role in cell cycle/growth	Functional evidence	Observed phenotype	Cell cycle phase affected	Organism	Key references
CDC5	AT1G09770	CELL DIVISION CYCLE 5	Core MAC/NTC-associated splicing factor that regulates genome-wide AS. Loss of CDC5 function causes widespread AS defects, predominantly exon skipping and intron retention, indicating a central role in splice-site selection and efficient intron removal.	Required for early embryonic cell division, G2/M transition, mitotic progression, and shoot apical meristem (SAM) maintenance. *AtCDC5* depletion reduces *CDKB1;1* expression, promotes endoreduplication, and disrupts WUS–CLV and STM pathways.	T-DNA mutant and RNAi knockdown assays; RNA-seq and differential AS analysis; qRT-PCR splicing validation; protein–protein interaction assays; flow cytometry; histological analysis; *in situ* hybridization; CLV3::GUS reporter analysis; STM overexpression rescue.	Loss-of-function causes embryonic lethality and zygote-stage arrest. RNAi plants show reduced growth, shorter roots, smaller rosettes, impaired SAM, defective inflorescence formation, reduced *CDKB1;1* expression, increased ploidy, enlarged cells, enhanced endoreduplication; genome-wide AS defects, and impaired splicing of *FLC, SEF* and *MAF* transcripts.	G2/M transition; endocycle/endoreduplication control	*Arabidopsis thaliana*	Palma et al., 2007; Lin et al., 2007; Xin et al., 2025
SCI1	AT1G79200	Stigma/style cell cycle inhibitor 1	Interact with NtCDKG;2, NtCyclin L, NtRH35, and spliceosome-related proteins such as U2A′, NKAP, and CACTIN. SCI1 and its partners co-localize in splicing speckles, suggesting a role in spliceosome-associated RNA processing.	Acts as a negative regulator of cell proliferation in reproductive tissues, controling cell number in the stigma/style and upper pistil and is expressed in floral meristematic cells. Its expression is regulated by auxin-related pathways and by *WUSCHEL* and *AGAMOUS* genes, linking developmental signaling to proliferation control.	RNAi knockdown; overexpression and T-DNA mutant analysis; histological analysis; cell number quantification; qRT-PCR; *in situ* hybridization; promoter-GFP analysis; Y1H; EMSA; luciferase reporter assay; semi-*in vivo* pull-down; mass spectrometry; Y2H; BiFC; protein co-localization in splicing speckles.	RNAi lines and T-DNA insertion mutants show enlarged upper pistil tissues due to increased cell number, whereas *SCI1* overexpression reduces organ size/cell number.	Not directly defined; controls cell proliferation/cell number	*Arabidopsis thaliana/Nicotiana tabacum*	DePaoli et al., 2011, 2014; Cruz et al., 2021; Pinoti et al., 2024
PRMT5	AT4G31120	Protein arginine methyltransferase 5	Regulates pre-mRNA splicing and AS through methylation of spliceosome- and RNA-processing-associated proteins. Loss of PRMT5 causes broad splicing defects, mainly intron retention, weak 5′ donor splice-site misrecognition, impaired NTC recruitment, and reduced splicing fidelity.	Promotes vegetative growth, root elongation, flowering-time control, fertility, and developmental progression, associated with altered FLC regulation and defective RNA processing.	Methyltransferase assays; symmetric arginine dimethylation detection; T-DNA/CRISPR mutant analysis; RNA-seq/AS profiling; intron-retention and weak 5′ donor splice-site analyses; PRP8 suppressor screen; Prp8/Brr2 IP-MS; Col-0/Ler donor splice-site variation analysis.	*prmt5* mutants show slow vegetative growth, shorter primary roots, smaller cotyledons and rosettes, dark-green curled leaves, delayed flowering, reduced fertility, altered circadian rhythms, and broad AS defects. PRP8 suppressors partially rescue splicing and developmental defects, while PRMT5 loss increases accession-dependent splicing and developmental variation.	Not reported	*Arabidopsis thaliana*	Pei et al., 2007; Deng et al., 2010; Sanchez et al., 2010; Hernando et al., 2015; Deng et al., 2016; Beckel et al., 2025
MAC3A/B	AT1G04510/AT2G33340	MOS4-Associated Complex (MAC)	Core MAC/Prp19C-associated proteins linked to spliceosome function. Loss of MAC3A/MAC3B causes global intron-retention defects, supporting a role in efficient pre-mRNA splicing and spliceosome-associated AS regulation. Direct target-specific AS regulation has not been demonstrated.	Regulate developmental growth, organ size, flowering time, fertility, and endoreduplication. Control organ growth through the UBP14/DA3–CDKB1;1 module, promoting CDKB1;1 degradation and modulating ploidy levels.	T-DNA mutant analysis; RNA-seq and intron-retention analysis; pri-miRNA and miRNA assays; protein–protein interaction assays; DCL1/HYL1 localization assays; U-box decoy screen; EMS suppressor screen; ubiquitination assays; flow cytometry; cell-size and organ-size measurements.	*mac3a mac3b* mutants show reduced plant size, shorter roots, shorter siliques, increased seed abortion, delayed flowering, altered miRNA accumulation, broad intron-retention defects, reduced ploidy levels, lower endoreduplication index, reduced cotyledon cell size, and fewer highly branched trichomes.	Mainly endoreduplication/endocycle control; indirect connection to G2/M through CDKB1;1 stability	*Arabidopsis thaliana*	Jia et al., 2017; Li et al., 2018; Feke et al., 2020; Guo et al., 2024
CDKC;2	AT5G64960	Cyclin-dependent kinase C;2	Colocalizes with spliceosomal components and modulates the nuclear distribution of SR/splicing factors in a transcription- and kinase-dependent manner, suggesting a role in coupling Pol II transcription with spliceosome-associated RNA processing.	Acts as a negative regulator of cell division and organ growth. Loss of CDKC;2 increases cell number in leaves and stems, alters cell layers, affects expression of cell cycle genes, reduces stomatal density, delays growth. Contributes to flowering time and circadian regulation through Pol II CTD phosphorylation.	GFP colocalization and redistribution assays with spliceosomal components; Pol II CTD phosphorylation assays; T-DNA mutant analysis; histological and cell-number analyses; qRT-PCR of cell cycle genes.	T-DNA mutants show enlarged lateral organs, increased epidermal cell number, altered cell layer organization, smaller cells, reduced ploidy levels, altered expression of cell cycle genes, and delayed growth/flowering.	Associated with increased cell division, altered endoreduplication/ploidy, and transcriptional regulation of cell cycle genes	*Arabidopsis thaliana*	Barrôco et al., 2003; Kitsios et al., 2008; Zhao et al., 2017
CDKG;2	AT1G67580	Cyclin dependent kinase (class G) with putative PLTSLRE cyclin binding motifs	Regulates temperature-dependent AS, including *FLM* AS and the *CDKG1–ATU2AF65A* splicing circuit. Acts with Cyclin L/CYCL1 and is associated with spliceosome-related proteins, supporting a role in spliceosome-associated RNA processing.	Promotes endoreduplication, ploidy level, and cell growth through the UBP14/DA3–CDKB1;1–CDKG2 module. Also regulates flowering time and reproductive development through AS-dependent pathways.	RT-qPCR assays; T-DNA insertion mutants; EMS suppressor alleles; protein interaction assays; Y2H; pull-down; BiFC; nuclear colocalization in splicing speckles; genetic analysis; flow cytometry; cell-size and organ-size measurements.	*cdkg2* mutants show altered *FLM* AS, early flowering, altered temperature-dependent AS responses, and reduced endoreduplication/cell growth in genetic backgrounds affecting the UBP14/DA3–CDKB1;1 pathway.	Endoreduplication/endocycle control.	*Arabidopsis thaliana/Nicotiana tabacum*	Ma et al., 2015; Cavallari et al., 2018; Nibau et al., 2020a; Jiang et al., 2022; Pinoti et al., 2024
Cyclin L/MOS12	AT2G26430	Cyclin with SR (serine/arginine-rich) type domains; MAC-associated splicing regulator	Regulates AS/splicing of selected transcripts and associates with the MAC spliceosome-related complex. Also participates with CDKG1/CDKG2 in temperature-dependent AS circuits controlling *CDKG1, ATU2AF65A*, and *FLM*. Interacts with spliceosome-associated proteins such as U2A′, NKAP, and CACTIN,	Required for normal plant growth and development. Partial loss of function causes developmental defects, whereas the null allele is lethal.	EMS partial loss-of-function mutant; T-DNA null allele; complementation with MOS12-GFP; RT-PCR splicing assays; co-immunoprecipitation; mutant phenotyping.	T-DNA mutant plants show altered splicing of *SNC1* and *RPS4*, reduced *SNC1* transcript/protein accumulation, smaller/lighter leaves, and suppression of *snc1* autoimmune phenotypes. The *mos12–2* null allele is homozygous lethal.	Not reported	*Arabidopsis thaliana/Nicotiana tabacum*	Xu et al., 2012; Cavallari et al., 2018; Nibau et al., 2020b; Pinoti et al., 2024
SR45	AT1G16610	Serine/arginine-rich (SR) protein 45	Essential splicing factor and regulate alternative splicing of other SR genes. Interacts with U1-70K, SR33, and AFC2, linking it to early spliceosome assembly and splice-site regulation.	Regulates multiple developmental and growth-related processes, including flowering time, leaf and flower development, root growth, early seedling development, glucose/ABA signaling, and metabolic gene expression. Subtle effects on cell division and cell expansion during leaf development.	Protein–protein interaction assays; *in vitro* splicing complementation; T-DNA mutant analysis; RT-PCR; promoter/activity data; glucose/ABA response assays; gene expression analysis.	T-DNA mutants show pleiotropic developmental defects, including delayed flowering, reduced plant size, slower root growth, narrow/curly leaves, altered petal and stamen number, impaired cotyledon greening and expansion under glucose, reduced hypocotyl elongation, ABA hypersensitivity, altered ABA accumulation, and altered expression of photosynthetic, metabolic, and ABA-related genes.	Not reported	*Arabidopsis thaliana*	Golovkin and Reddy, 1999; Ali et al., 2007; Carvalho et al., 2010
SC35-like (SCL)	AT5G64200	Serine/Arginine-rich splicing factors (ortholog of human splicing factor SC35)	Regulate pre-mRNA splicing and alternative splicing of a subset of genes. Localize to nuclear speckles, interact with U1-70K and U2AF65a, and affect intron retention, exon skipping, and alternative splice-site usage.	Required for normal developmental growth.	T-DNA insertion mutants; CRISPR/Cas9-generated multiple mutants; RNA-seq; RT-PCR splicing validation; GUS reporter analysis; YFP/CFP subnuclear localization; colocalization assays; yeast two-hybrid interaction assays.	*sc35-scl* quintuple mutant exhibits serrated rosette leaves, smaller late-stage rosette leaves, delayed flowering, increased rosette leaf number, shorter roots, and abnormal silique phyllotaxy.	Not reported	*Arabidopsis thaliana*	Golovkin and Reddy, 1999; Yan et al., 2017
SmD1b	AT4G02840	Small nuclear ribonucleoprotein D1	Core Sm protein associated with spliceosomal snRNPs that regulates alternative splicing, particularly by limiting intron retention in endogenous transcripts.	Required for developmental growth and viability, affecting rosette size, leaf morphology, flowering time, and embryo development.	PTGS-deficient mutant screen; T-DNA mutant and genetic interaction analyses; genomic/GFP complementation; confocal localization and colocalization with SR34; RT-PCR and RNA-seq analysis of intron retention and alternative splicing.	T-DNA mutants show reduced stature, serrated leaves, early flowering, impaired PTGS, and intron retention in endogenous transcripts. SmD1 complementation restores development and PTGS, while *smd1a smd1b* double mutants are embryo-lethal.	Not reported	*Arabidopsis thaliana*	Elvira-Matelot et al., 2016; Bazin et al., 2023
SmD3	AT1G20580	Small nuclear ribonucleoprotein D3	Core snRNP protein required for efficient pre-mRNA splicing and essential for splicing of primary transcripts.	Required for developmental growth, affecting root growth, leaf venation, trichome branching, flowering time, floral organ number, silique development, and life-cycle progression.	T-DNA knock-out mutants; SmD3-b complementation; RT-PCR; microarray profiling; phenotypic analysis.	*smd3-b* mutants show reduced root growth, defective leaf venation, abnormal trichome branching, delayed flowering, altered floral organ number, abnormal siliques, aborted ovules, reduced seed number, and delayed life-cycle.	Not reported	*Arabidopsis thaliana*	Swaraz et al., 2011
U1-70K	AT3G50670	U1 small nuclear ribonucleoprotein (snRNP)-70K	Core U1 snRNP-specific protein required for early spliceosome assembly, contributing to 5′ splice-site recognition through U1 snRNA association and interactions with plant SR proteins.	Required for normal plant development, particularly floral organ development. Tissue-specific antisense suppression of U1-70K in petals and stamens disrupts floral organ formation.	RT-PCR/RNA blot; U1 snRNA-binding and immunodetection assays; yeast two-hybrid, blot-overlay and coprecipitation assays; AP3 promoter-driven antisense suppression with floral phenotypic and AP3/PI expression analyses.	Antisense suppression of U1-70K causes rudimentary or arrested petals and stamens, severe floral organ defects, and male sterility in strong transgenic lines. U1-70K also produces full-length and truncated transcript isoforms through alternative splicing.	Not reported	*Arabidopsis thaliana*	Golovkin and Reddy, 1999, 2003
MAS2 (NKAP)	AT4G02720	MORPHOLOGY OF AGO1–52 SUPPRESSED2 (MAS2)	Regulates RNA processing/splicing fidelity by modulating AGO1 splice isoform ratios; *mas2* alleles suppress *ago1* missplicing phenotypes and MAS2 interacts with splicing- and ribosome biogenesis-related proteins.	Essential for plant development. T-DNA insertion alleles are embryonic lethal, and partial MAS2 silencing causes severe developmental defects	EMS suppressor alleles; positional cloning and sequencing; T-DNA insertion mutants; *amiR*-*MAS2* silencing; RT-qPCR; immunoblot; Y2H interaction assays; GFP localization; FISH; 45S rDNA methylation and NOR organization analyses.	*mas2* suppressor alleles modify *ago1* missplicing phenotypes. Null alleles are embryonic lethal, while *amiR*-*MAS2* plants show dwarfism, altered leaves, early flowering, reduced fertility, aborted seeds, and defects in 45S rDNA silencing/NOR organization.	Not reported	*Arabidopsis thaliana*	Sánchez-García et al., 2015; Cabezas-Fuster et al., 2022

Experimentally supported proteins involved in AS or RNA processing that also affect cell proliferation, endoreduplication, organ growth, developmental progression, fertility, or other cell cycle associated processes, highlighting candidate connections between splicing regulation and growth control in plants.

#### 4.2.1 Transcriptional and spliceosome-associated regulators

##### 4.2.1.1 CDC5: a conserved transcriptional and spliceosomal regulator

Plant CDC5 is a MYB-related transcription factor and a core component of the MAC complex, the plant ortholog of the conserved Prp19/NTC involved in spliceosome activation. Physical interaction studies identified CDC5 as a central MAC subunit associated with MOS4 and PRL1, supporting its connection with the spliceosome and pre-mRNA processing machinery (Palma et al., 2007). Consistently, transcriptomic analyses of MAC mutants, including *mac7*, *mac3a mac3b*, and *prl1 prl2*, revealed widespread intron retention and splice-site defects, demonstrating that MAC integrity is required for efficient pre-mRNA splicing in plants (Jia et al., 2017; Li et al., 2018). More recently, genome-wide analyses of *cdc5* mutants confirmed that CDC5 itself regulates AS, affecting intron retention and splice-site selection events (Xin et al., 2025).

CDC5 also coordinates transcriptional and post-transcriptional regulation of flowering. It represses floral transition by activating *FLC* expression through association with *FLC* chromatin, increased RNA polymerase II enrichment, and interaction with the Paf1 complex. In parallel, CDC5 modulates the AS of flowering-related genes, including *FLC* (FLOWERING LOCUS C), *SEF* (SERRATED LEAVES AND EARLY FLOWERING), and *MAF* genes (MADS AFFECTING FLOWERING) (Xin et al., 2025).

AtCDC5 is also essential for cell cycle progression and plant development. It is predominantly expressed in proliferating tissues, and complete loss of function causes embryonic lethality, with embryos arrested at the zygote stage and defective endosperm division (Lin et al., 2007). The involvement of AtCDC5 in the G2/M transition is supported by RNAi lines showing increased nuclear ploidy levels and enlarged epidermal cells, suggesting impaired mitotic progression and a shift toward endoreduplication. These phenotypes are associated with reduced *CDKB1;1* expression, a key regulator of G2/M progression, and altered expression patterns of cell cycle markers such as *CYCB1* and *HISTONE 4* (**Figure 3**). AtCDC5 is additionally required for shoot apical meristem maintenance, as its depletion causes abnormal growth, impaired apical dominance, and reduced expression of *WUSCHEL (WUS)* and *SHOOT MERISTEMLESS (STM)*. Together, these findings identify CDC5 as a multifunctional regulator linking transcription, pre-mRNA splicing, cell cycle progression, and developmental maintenance (**Figure 3**).

##### 4.2.1.2 SCI1: linking cell proliferation to spliceosome-associated complexes

SCI1 is a nuclear, tissue-specific regulator that restricts cell proliferation during reproductive organ development in *Nicotiana tabacum* and Arabidopsis (DePaoli et al., 2011, 2014). Loss- and gain-of-function analyses showed that *SCI1* controls upper pistil size mainly by modulating cell number: *SCI1* silencing or mutation increases stigma/style or upper pistil tissues, whereas *SCI1* overexpression reduces organ size (DePaoli et al., 2011, 2014). Its expression in floral meristems and meristematic cells, together with putative cyclin-interaction motifs, functional similarities to plant CDK inhibitors, and regulation by auxin-related pathways and floral meristem regulators such as WUS and AGAMOUS, places SCI1 as a developmental effector linking proliferative control, differentiation, and reproductive organ growth (DePaoli et al., 2011, 2014; Cruz et al., 2021; Strini et al., 2022).

A major recent advance was the identification of SCI1 as a protein physically associated with spliceosome-related complexes. SCI1 interacts with NtCDKG;2, NtCyclin L, and the DEAD-box RNA helicase NtRH35, and these proteins co-localize in nuclear splicing speckles and the nucleolus (Pinoti et al., 2024; **Figure 3**). NtRH35 is particularly relevant because its human homolog, DDX41/Abstrakt, links spliceosome function to transcriptional elongation, genome stability, and cell proliferation (Bessonov et al., 2008; Polprasert et al., 2015; Tsukamoto et al., 2020; Shinriki et al., 2022; Weinreb et al., 2022; Pinoti et al., 2024). The SCI1-associated network includes spliceosome-related proteins such as U2A′, NKAP, and CACTIN, further supporting a connection between SCI1-dependent proliferation control and pre-mRNA processing machinery (Pinoti et al., 2024). This is particularly relevant because direct interaction-based links between tissue-specific regulators of floral cell proliferation and spliceosome-associated components are scarce in plants. Together, these findings place SCI1 as an emerging tissue-specific regulator of proliferation that interfaces with spliceosome-associated protein networks, suggesting a possible route by which RNA-processing machinery may contribute to reproductive organ growth.

#### 4.2.2 Multifunctional MAC components integrating splicing and cell cycle associated growth

MAC3A and MAC3B are conserved U-box E3 ubiquitin ligases and core subunits of the MAC, the plant counterpart of the spliceosome-associated NTC. Transcriptomic analyses of the *mac3a mac3b* double mutant revealed widespread intron-retention defects, supporting a role for MAC3A and MAC3B in efficient intron removal and AS regulation (Jia et al., 2017). MAC3A/B also contribute to microRNA biogenesis by associating with pri-miRNAs and the DCL1 complex, thereby promoting pri-miRNA stability and processing (Li et al., 2018).

Beyond RNA metabolism, MAC3A/B connect MAC function with ubiquitin-mediated developmental control. They physically interact with and ubiquitinate UBIQUITIN-SPECIFIC PROTEASE 14/DA3 (UBP14/DA3), modulating the UBP14/DA3–CDKB1;1 pathway that controls ploidy level, endoreduplication, cell expansion, and organ size (Guo et al., 2024). Thus, MAC3A/B act upstream of a cell cycle-associated growth module, linking E3 ligase activity to endocycle regulation rather than direct mitotic proliferation (**Figure 3**).

Consistently, *mac3a mac3b* mutants display pleiotropic developmental phenotypes, including reduced plant size, shorter roots, shortened siliques, increased seed abortion, altered trichome branching, delayed flowering, reduced endoreduplication index, lower ploidy levels, and impaired cotyledon cell expansion (Li et al., 2018; Feke et al., 2020; Guo et al., 2024). Together, MAC3A/B emerge as multifunctional MAC components that couple spliceosome-associated RNA processing with ubiquitin-mediated control of endocycle-dependent organ growth.

#### 4.2.3 Cyclin-dependent kinases and cyclins associated with splicing

Among the non-canonical Arabidopsis CDK/cyclin-associated factors, CDKC;2, CDKG1, CDKG2, and CYCL1/MOS12 link spliceosome-associated RNA processing with developmental growth and proliferative processes (Vandepoele et al., 2002; Menges et al., 2005; De Veylder et al., 2007).

CDKC;2 provides an important link between Pol II-dependent RNA processing, spliceosome organization, and growth (**Figure 3**). As a Pol II CTD kinase, CDKC;2 contributes to transcriptional elongation and co-transcriptional RNA processing, and colocalizes with spliceosome-associated proteins in nuclear speckles (Barrôco et al., 2003; Cui et al., 2007; Kitsios et al., 2008). Although direct target-specific AS regulation by CDKC;2 has not been demonstrated, *cdkc;2* mutants show increased cell division during leaf development, altered cell number and cell size, reduced ploidy, and altered expression of cell cycle-related genes (Zhao et al., 2017).

CDKG2 provides the clearest functional connection between AS and endocycle-dependent growth. Together with CYCL1, CDKG2 regulates temperature-dependent AS of *FLOWERING LOCUS M* (*FLM*), thereby influencing flowering time, and contributes to the CDKG1–ATU2AF65A splicing circuit (Cavallari et al., 2018; Nibau et al., 2020a). CDKG2/SUD6 also promotes endoreduplication, ploidy level, and cell growth through the UBP14/DA3–CDKB1;1–CDKG2 pathway (Jiang et al., 2022; **Figure 3**). Thus, although CDKG2 is not a canonical mitotic CDK, it represents a key non-canonical kinase at the interface between AS-associated regulation, endocycle control, and developmental growth. CDKG1, in turn, connects splicing regulation with fertility, meiotic recombination, and reproductive development. It regulates *CalS5* splicing, pollen wall formation, and male fertility, and participates in a temperature-sensitive AS circuit involving CDKG1 itself and the spliceosomal factor ATU2AF65A (Huang et al., 2013b; Cavallari et al., 2018).

CYCL1 acts as a spliceosome-associated protein rather than as a canonical cell cycle cyclin. MOS12 associates with the MAC and is required for the proper splicing of selected transcripts, including SNC1 and RPS4 (Xu et al., 2012). Partial loss of MOS12 alters splicing and development, whereas the null allele is lethal, indicating an essential role in plant growth and viability. CYCL1 also acts with CDKG1 and CDKG2 in temperature-dependent AS circuits affecting CDKG1, ATU2AF65A, and FLM (Cavallari et al., 2018; Nibau et al., 2020b).

Together, these findings define a non-canonical CDK/cyclin module that integrates Pol II-dependent RNA processing, AS, endoreduplication, reproductive development, and growth, with CDKG2 currently providing the clearest functional bridge to endocycle-dependent growth.

#### 4.2.4 Spliceosome-associated regulators involved in developmental growth

For the factors discussed in Sections 4.2.4 -4.2.66, available evidence primarily supports roles in RNA processing and developmental growth, whereas direct regulation of defined cell cycle phases has not been demonstrated.

##### 4.2.4.1 MAS2/NKAP: an RNA-processing and nucleolar regulator linked to developmental growth

MAS2 encodes the putative Arabidopsis ortholog of NKAP (NF-κB activating protein), a conserved eukaryotic protein associated with transcriptional repression and RNA splicing in animals. In Arabidopsis, MAS2 functions as an essential nuclear protein involved in RNA processing, 45S rDNA regulation, and nucleolar organization (**Figure 3**). Sánchez-García et al. (2015) linked MAS2 to splicing fidelity by showing that *mas2* alleles alter AGO1 splice isoform ratios, suppress *ago1* missplicing phenotypes, and interact with RNA-processing and ribosome biogenesis factors.

MAS2 also localizes to nucleolar organizer regions (NORs), where it contributes to the epigenetic silencing of 45S rDNA. Partial MAS2 depletion causes hypomethylation of 45S rDNA promoters, NOR decondensation, increased rDNA transcription, and accumulation of unprocessed pre-rRNAs (Sánchez-García et al., 2015). Complete MAS2 loss is embryonic lethal, whereas partial depletion causes reduced growth, serrated leaves, early flowering, reduced fertility, and shorter siliques. These phenotypes indicate that MAS2 is essential for plant viability and developmental growth, although direct regulation of a specific cell cycle phase has not been demonstrated.

More recently, MAS2 was further placed within the Arabidopsis missplicing suppressor framework by the finding that PRP8/MAS5 alleles increase splicing fidelity by reducing the use of novel splice sites (Cabezas-Fuster et al., 2022). In parallel, the identification of NKAP in SCI1-associated spliceosome-related networks suggests that MAS2/NKAP-like proteins may participate in RNA-processing modules linked to developmental regulation (Pinoti et al., 2024). Thus, MAS2 is best viewed as an essential RNA-processing and nucleolar regulator with indirect links to plant growth and proliferation, rather than as a validated cell cycle regulator.

##### 4.2.4.2 SR45 and SCL proteins: SR-mediated splicing regulation in plant development

Among the stress-responsive splicing regulators summarized in **Table 1**, SR proteins are particularly relevant because their mutant phenotypes combine altered environmental responses with broad defects in development and RNA processing. Their potential connections with cell proliferation and cell cycle-associated growth are further examined in this section and summarized in **Table 3**, with particular emphasis on SR45 and SC35/SCL proteins.

In plants, the SR family is considerably expanded compared with animals, comprising 19 members in Arabidopsis and 23 in rice (Golovkin and Reddy, 1996; Kalyna and Barta, 2004; Iida and Go, 2006; Isshiki et al., 2006). Several of these proteins also interact with the U1-70K spliceosomal factor, and some represent plant-specific subfamilies with no clear orthologs in metazoans (Golovkin and Reddy, 1998, 1999; Reddy, 2007).

SR45 was initially identified as a U1-70K interacting factor in a yeast two-hybrid screen (Golovkin and Reddy, 1999). SR45 colocalizes with U1-70K and SR1 in nuclear speckles displays general splicing activity *in vitro* (Ali et al., 2003; Golovkin and Reddy, 1999). SR45 physically associates with spliceosomal proteins and contributes to early spliceosome assembly and splice-site recognition (**Figure 3**). Loss of SR45 function results in widespread AS defects, particularly affecting other SR protein transcripts and altering the balance between productive and nonproductive isoforms (Ali et al., 2007; Carvalho et al., 2010). These defects are accompanied by broad vegetative and reproductive defects (**Table 3**). Altered leaf cell expansion and increased stomatal number suggest that SR45 may indirectly influence cell expansion and proliferation during organ development, although direct control of a specific cell cycle phase has not been demonstrated (Ali et al., 2007; **Figure 3**). In addition, *sr45–1* seedlings display hypersensitivity to glucose and elevated ABA levels (**Table 1**), suggesting that SR45 integrates splicing regulation with hormonal and metabolic signaling pathways during development (Carvalho et al., 2010).

Arabidopsis SC35 belongs to the conserved SC35-related subfamily of SR proteins and contains the canonical RRM and RS domains (Golovkin and Reddy, 1999; Yan et al., 2017). SC35 and SC35-like proteins localize to nuclear speckles and physically associate with spliceosomal components, including U1-70K and U2AF65a, supporting their role in spliceosome-associated RNA processing. Depletion of SC35/SCL proteins causes widespread splicing defects, including intron retention, exon skipping, and altered splice-site selection in multiple endogenous transcripts. The *sc35-scl* quintuple mutant exhibits developmental abnormalities, including serrated rosettes, delayed flowering, shorter roots, and abnormal silique phyllotaxy (Yan et al., 2017). Transcriptomic analyses further showed that SC35/SCL proteins regulate both AS and the expression of a subset of genes, including genes involved in flowering regulation. Together, these findings establish SR45 and SC35/SCL proteins as important regulators of splice-site selection, transcript isoform balance, and plant development.

#### 4.2.5 Core spliceosomal components and snRNP-associated proteins

As discussed in Section 3.2, mutations affecting core spliceosomal components and snRNP-associated proteins frequently alter plant responses to environmental stress, demonstrating that spliceosome integrity is required for the accurate processing of stress-responsive transcripts. These factors also perform broader roles in plant development. Studies of U1-70K, SmD1, and SmD3 further show that disruption of snRNP function causes severe defects, including impaired organogenesis, reduced fertility, abnormal growth, and embryonic lethality. Although these proteins have not been directly linked to specific cell cycle phases, their phenotypes support an indirect connection between spliceosome integrity, environmental responsiveness, and growth regulation.

##### 4.2.5.1 U1-70K: early spliceosome assembly and floral organ development

U1-70K is a core U1 snRNP component involved in early spliceosome assembly and 5′ splice-site recognition in plants. In Arabidopsis, U1-70K is encoded by a single gene that undergoes AS and produces distinct transcript isoforms (**Figure 3**). The functional full-length U1-70K protein associates with U1 snRNA and physically interacts with multiple plant SR proteins, including SRZ21, SRZ22, SR33, and SR45, supporting its role in early spliceosome assembly and splice-site definition (Golovkin and Reddy, 1996, 1998, 1999). Targeted suppression of U1-70K expression in floral organs using APETALA3 promoter-driven antisense constructs resulted in arrested petal and stamen development, rudimentary floral organs, and male sterility in severe transgenic lines (Golovkin and Reddy, 2003).

##### 4.2.5.2 Sm proteins: core snRNP components required for RNA processing and developmental growth

Sm proteins are highly conserved spliceosomal proteins found across eukaryotes (Séraphin, 1995). The canonical Sm proteins (SmB, SmD1, SmD2, SmD3, SmE, SmF, and SmG) share conserved Sm1/Sm2 motifs and assemble into the core Sm ring of U1, U2, U4, and U5 snRNPs, a structural organization supported by biochemical and crystallographic studies (Kambach et al., 1999). Related LSM proteins form distinct Sm-like complexes (**Table 1**), including LSM2–8, which associates with the U6 snRNP during pre-mRNA splicing, and LSM1–7, which functions mainly in cytoplasmic mRNA decapping and decay (Séraphin, 1995).

In Arabidopsis, SmD1 is encoded by two closely related genes, SmD1a and SmD1b, which act redundantly, although SmD1b is functionally predominant. SmD1b localizes to the nucleus, including nucleoli and nucleoplasmic speckles, where it colocalizes with the splicing-related SR protein SR34, supporting its association with spliceosome-related nuclear domains. Loss of SmD1b affects endogenous transcript splicing, particularly by increasing intron retention, and RNA-seq analyses further support its role in AS regulation (Elvira-Matelot et al., 2016; Bazin et al., 2023; **Figure 3**). Consistently, *smd1b* mutants show reduced growth, serrated leaves, altered flowering time, and splicing defects, whereas *smd1a smd1b* double mutants are embryo-lethal. Additional studies showed SmD1 as targeted by the *Meloidogyne incognita* effector 18 (MiEFF18) and is required for proper giant cell formation, linking SmD1-dependent splicing regulation to infection-induced cellular reprogramming (Mejias et al., 2021, 2022).

SmD3 is encoded in Arabidopsis by two related genes, SmD3-a and SmD3-b, with SmD3-b playing the predominant functional role. Loss of SmD3-b blocks or reduces splicing of selected endogenous transcripts and causes pleiotropic developmental defects, including reduced root growth, delayed flowering, altered leaf venation, abnormal trichome branching, floral organ defects, shorter siliques, aborted ovules, and reduced seed number (Swaraz et al., 2011). Biochemical evidence from mammalian cells further supports the conserved role of SmD3 in snRNP assembly and maturation (Khusial et al., 2005).

#### 4.2.6 Post-translational regulation of the spliceosome

##### 4.2.6.1 PRMT5-mediated regulation of spliceosome fidelity and developmental robustness

Building on the essential developmental roles of Sm proteins described above, PRMT5, also known as SKB1 in Arabidopsis, regulates snRNP function through symmetric dimethylation of spliceosomal proteins, including SmD1, SmD3, and LSM4. Among the nine Arabidopsis PRMT genes, PRMT5 is the best-characterized member associated with pre-mRNA splicing, alternative splicing, and development (Pei et al., 2007; Deng et al., 2010; Sanchez et al., 2010; Hernando et al., 2015).

Loss of PRMT5 reduces arginine dimethylation of Sm proteins and causes widespread splicing defects, particularly intron retention and impaired recognition of weak 5′ splice sites (**Figure 3**). PRMT5 also promotes the recruitment of the NTC and catalytic-step factors during spliceosome activation, as supported by the partial suppression of *prmt5* molecular and developmental defects by neomorphic PRP8 mutations (Deng et al., 2010, 2016; Sanchez et al., 2010; Hernando et al., 2015). Beyond this basal role in spliceosome fidelity, PRMT5 buffers the effects of natural genetic variation on splice-site selection. Accordingly, its loss in genetically divergent Arabidopsis accessions accentuates accession-dependent splicing differences, particularly at polymorphic 5′ splice sites, and increases variation in leaf morphology, flowering time, and circadian traits (Beckel et al., 2025; **Figure 3**). Consistent with these molecular functions, *prmt5* mutants exhibit reduced growth, shorter roots, curled dark-green leaves, delayed flowering, reduced fertility, and circadian defects. Delayed flowering is associated with increased FLC expression and is partially rescued in *prmt5 flc* double mutants, supporting a role for PRMT5 in the autonomous flowering pathway (Pei et al., 2007; Sanchez et al., 2010; Beckel et al., 2025; **Table 3**). Together, these findings establish PRMT5 as a post-translational regulator that connects arginine methylation of spliceosomal proteins with splicing fidelity, developmental stability, and plant growth.

## 5 Potential roles of chromatin-associated splicing factors in replication stress and genome stability

A major question in plant biology is how environmental signals are translated into coordinated changes in chromatin organization, RNA processing, and cell cycle progression (Jia et al., 2020). Rather than operating as independent regulatory layers, increasing evidence suggests that chromatin dynamics, AS, and cell cycle control are functionally interconnected processes that collectively determine plant growth and stress adaptation (Petasny et al., 2021). Within this framework, chromatin not only regulates whether genes are transcribed, but also influences how nascent transcripts are processed and how developmental programs are adjusted in response to environmental challenges. This integration is particularly relevant in plants, where adverse conditions frequently trigger transitions from active proliferation to growth restraint, endoreduplication, cellular repair, or developmental reprogramming (Ullah et al., 2018).

Several studies have demonstrated that chromatin modifications actively shape AS outcomes in plants. In Arabidopsis, temperature-responsive alternatively spliced genes are enriched in H3K36me3, and disruption of this histone mark alters both temperature-dependent AS patterns and flowering responses, highlighting the contribution of chromatin-mediated splicing regulation to developmental plasticity (Pajoro et al., 2017). Likewise, stochastic changes in DNA methylation can alter nucleosome occupancy and reshape cold-responsive AS profiles in genes associated with RNA metabolism, hormone signaling, and stress adaptation (Jabre et al., 2022). More recently, the H3K36me3 reader MORF4-RELATED GENE 2 (MRG2) was shown to interact with the exon junction complex component eukaryotic translation initiation factor 4A-3 (eIF4A3), directly coupling chromatin recognition to co-transcriptional AS of circadian clock genes (Huang et al., 2025). Together, these findings indicate that chromatin marks do not merely reflect transcriptional activity but actively participate in determining AS outputs.

Consistent with this view, AS frequently occurs co-transcriptionally, creating opportunities for chromatin states to influence splice-site selection through effects on RNA polymerase II dynamics and the recruitment of splicing-associated factors (Luco et al., 2011; Naftelberg et al., 2015). Increasing evidence suggests that these mechanisms operate in concert, allowing environmental cues to simultaneously influence transcription, chromatin accessibility, and RNA processing. Such coordination has important implications for plant growth because many stress-responsive pathways ultimately converge on developmental programs and cell cycle regulation.

**Figure 4:**
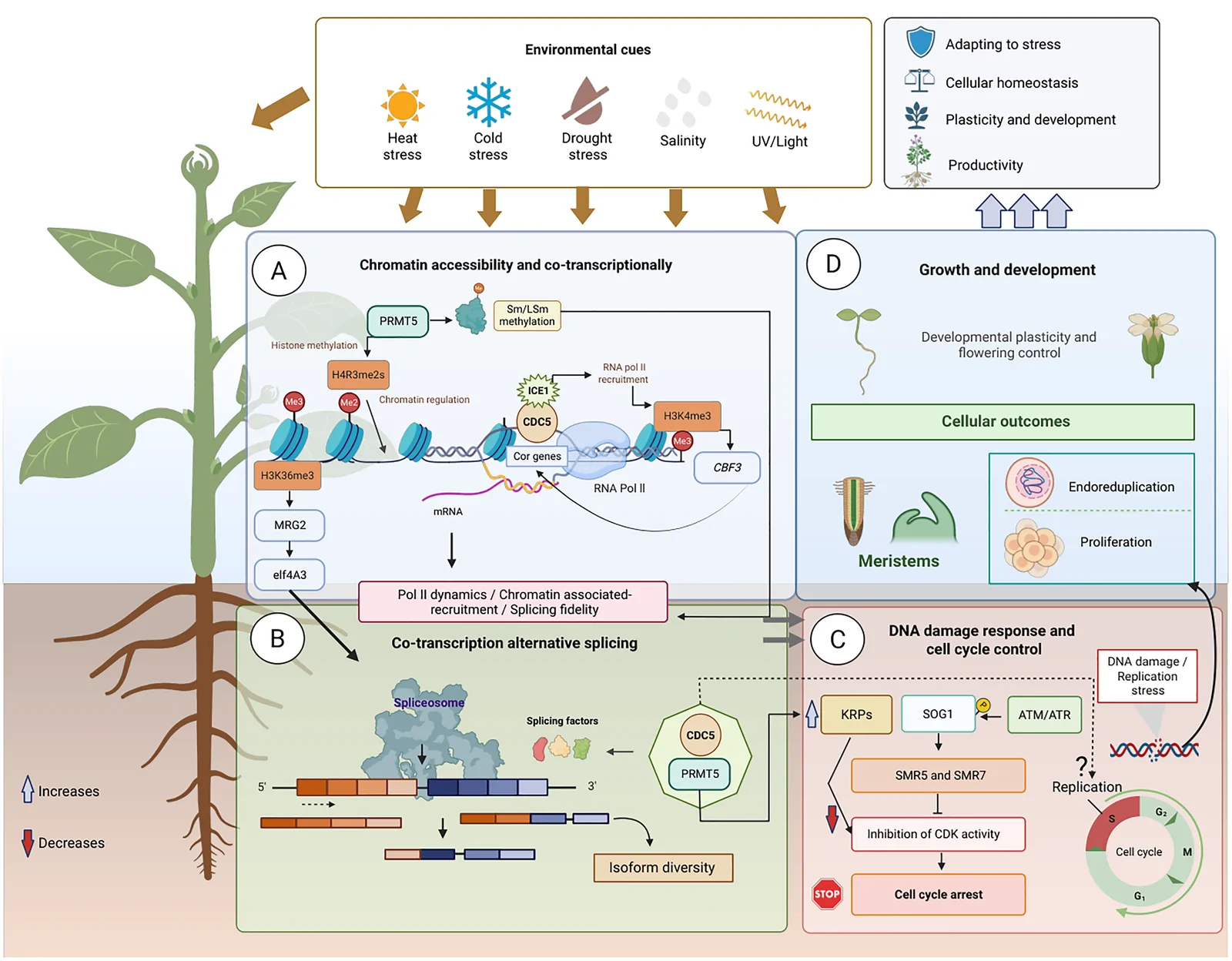
Chromatin–AS crosstalk coordinates cell cycle progression and stress adaptation in plants. Environmental cues, including heat, cold, drought, salinity, and UV/light, trigger coordinated changes in chromatin organization, transcriptional activity, RNA processing, and cell cycle progression. **(A)** Protein Arginine Methyltransferase 5 (PRMT5/SKB1) connects histone arginine methylation and Sm/LSm (Sm and Like-Sm) protein methylation with chromatin regulation and splicing fidelity whereas Cell Division Cycle 5 (CDC5) cooperates with INDUCER OF CBF EXPRESSION 1 (ICE1) to promote RNA polymerase II (RNAPII) recruitment, enhance trimethylation of histone H3 lysine 4 (H3K4me3), and activate *C-REPEAT BINDING FACTOR 3* (*CBF3*) cold-responsive. **(B)** These chromatin modifications and transcriptional mechanisms converge with co-transcriptional AS to generate transcript isoform diversity, thereby modulating genes involved in stress responses, development, genome stability, and growth regulation. The MORF4-Related Gene 2 (MRG2) and Eukaryotic Translation Initiation Factor 4A-3 (eIF4A3) module links histone methylation to recruitment of RNA-processing machinery. Furthermore, PRMT5 indirectly promotes cell proliferation by limiting the activity of negative regulators of the cell cycle by Kip-Related Proteins (KRPs). **(C)** In parallel, environmental stress can induce DNA damage and replication stress, leading to the activation of Ataxia Telangiectasia Mutated (ATM) and ATM and Rad3-Related (ATR), which phosphorylate and activate Suppressor of Gamma Response 1 (SOG1). Signaling and Cyclin-Dependent Kinase (CDK) inhibitors such as SIAMESE-RELATED 5 and 7 (SMR5 and SMR7), leading to reduced CDK activity and cell cycle arrest. RNA-processing factors such as CDC5 and PRMT5 represent candidate links between splicing, genome surveillance, and cell cycle control through the regulation of replication. **(D)** The integration of chromatin regulation, alternative splicing, and genome surveillance ultimately influences cell cycle progression, thereby affecting plant growth, development, and stress adaptation. Created in BioRender. Araújo-lopes, **(B)** (2026) https://BioRender.com/ia3fmhv.

Among the factors summarized in **Table 3**, CDC5 and PRMT5/SKB1 provide particularly compelling examples of this regulatory integration. CDC5, originally characterized as a spliceosome-associated protein and cell cycle regulator, has recently emerged as a chromatin-associated factor involved in cold adaptation (**Figure 4**). CDC5 cooperates with INDUCER OF CBF EXPRESSION 1 (ICE1) to promote *C-REPEAT BINDING FACTOR 3* (*CBF3*) expression (**Figure 4**) under low temperatures by facilitating RNA polymerase II occupancy and enhancing active chromatin marks, including H3K4me3 and histone H3 lysine 18 acetylation (H3K18ac), at the *CBF3* locus. These activities position CDC5 at the interface between chromatin remodeling, transcriptional activation, AS regulation, and developmental responses to environmental stress (Xin et al., 2024, 2025).

PRMT5/SKB1 further strengthens this concept because it simultaneously functions as a chromatin modifier and a key regulator of pre-mRNA splicing. In Arabidopsis, PRMT5 catalyzes symmetric dimethylation of histone H4 arginine 3 (H4R3me2s) while also controlling the splicing of hundreds of genes involved in flowering, circadian regulation, and stress responses (Pei et al., 2007; Deng et al., 2010). Loss of *PRMT5* function compromises shoot regeneration and alters expression of KRP genes, which encode CDKs inhibitors involved in cell cycle control (Liu et al., 2016). These observations place PRMT5 at a strategic position linking chromatin regulation, RNA processing, and developmental growth programs (**Figure 4**).

Despite these findings, direct evidence connecting chromatin-mediated AS regulation to cell cycle progression in plants remains remarkably limited. While PRMT5 plays important roles in epigenetic regulation and cell cycle related gene expression, its mechanistic involvement in DNA replication processes remains uncharacterized in plants. Interestingly, studies in mammalian systems suggest that such integration may occur. PRMT5 has been reported to interact with Minichromosome Maintenance complex 7 (MCM7), a highly conserved component of the MCM2–7 helicase complex required for DNA replication licensing during the G1/S transition (Li et al., 2021b). In human cells, MCM7 has also been shown to physically interact with the U2 snRNP component splicing factor 3B Subunit 3 (SF3B3), and depletion of either protein impairs pre-mRNA splicing efficiency and significantly increases cell death, highlighting the importance of MCM7/SF3B3-mediated splicing for cell survival (Chen et al., 2015). Together, these findings suggest a potential molecular link between DNA replication and RNA splicing machineries that may be evolutionarily conserved, although this remains to be demonstrated in plants.

These findings suggest a potential molecular connection between DNA replication machinery and the spliceosome, raising the possibility that analogous mechanisms may exist in plants. More broadly, they suggest that the functional interplay between RNA processing and cell cycle related processes may extend beyond developmental regulation to encompass the maintenance of genome integrity during DNA replication (Gill and Tuteja, 2010; Nimeth et al., 2020; Żabka et al., 2021). Because both chromatin organization and RNA processing influence replication dynamics, factors operating at the intersection of these processes are emerging as potential regulators of genome stability during replication (Żabka et al., 2021). Consistent with this view, evidence from plants suggests that components linking chromatin regulation and RNA processing contribute to DNA damage responses and the maintenance of meristematic activity under genotoxic stress. Among these factors, PRMT5/SKB1 provides one of the most compelling examples of how RNA-processing regulators can influence genome stability pathways.

In plants, the PRMT5/SKB1 complex provides one of the clearest examples of the association between RNA processing and genome stability maintenance (Li et al., 2016). Loss of PRMT5 function compromises the survival of root stem cells under genotoxic stress, and the sensitivity of *skb1* mutants is further exacerbated when combined with *atm* and *atr* mutations, suggesting that PRMT5 functionally cooperates with the DNA damage response (DDR) pathways (Li et al., 2016). These findings indicate that regulators operating at the interface between chromatin regulation and RNA processing may play important roles in maintaining proliferative tissues exposed to challenges that threaten genome integrity (**Figure 4**).

Additional evidence from animal systems further supports this possibility. In human cells, the Prp19/CDC5L complex is recruited to RPA-coated single-stranded DNA regions following genomic damage and contributes to ATR kinase activation, a central event in replication checkpoint signaling (Maréchal et al., 2014). Furthermore, depletion of Prp19 or CDC5L delays S-phase progression, impairs the recovery of collapsed replication forks, and compromises checkpoint activation under conditions of replication stress (Zhang et al., 2009; Maréchal et al., 2014; Saldivar et al., 2017). Given that the Arabidopsis MAC is functionally related to the Prp19 complex and that CDC5 is the plant homolog of human CDC5L (**Table 2**), these observations raise the possibility that similar connections between RNA processing and replication checkpoint control may also exist in plants (**Figure 4**). If so, such mechanisms would likely intersect with the major signaling pathways that coordinate replication stress responses and genome surveillance in plants.

In response to replication stress, SOG1 orchestrates a broad transcriptional program that induces DNA repair genes, negative regulators of cell cycle progression such as *SIAMESE-RELATED 5* (*SMR5*) and *SIAMESE-RELATED* 7 (*SMR7*), and additional components involved in preserving genome stability (Yoshiyama et al., 2009; Yoshiyama, 2015; Yi et al., 2014; Ogita et al., 2018; **Figure 4**). Because lesion recognition and repair occur within a chromatin context, activation of this pathway is accompanied by extensive chromatin remodeling, including γH2AX deposition (the phosphorylated form of the histone variant H2AX that serves as an early marker of DNA double-strand breaks), histone post-translational modifications, changes in DNA accessibility, and the recruitment of chromatin-remodeling complexes required to facilitate repair machinery access to damaged sites (Kim, 2019; Nisa et al., 2019).

Although these mechanisms have considerably advanced our understanding of replication stress signaling in plants, how chromatin regulators and splicing-associated factors integrate into these pathways remains largely unknown. Given their dual roles in epigenetic regulation, RNA processing, and growth- and proliferation-related processes, proteins such as CDC5 and PRMT5 emerge as particularly promising candidates for coordinating chromatin dynamics, alternative splicing, and checkpoint responses during replication stress (**Figure 4**). However, direct evidence is still lacking as to whether these factors influence ATM/ATR–SOG1 signaling or regulate the expression and processing of genes involved in replication checkpoint control in plants. Therefore, understanding how epigenetic and post-transcriptional mechanisms converge to modulate the replication stress response represents a major knowledge gap and an important frontier for future research in plant biology.

## 6 Future perspectives for the study of splicing

**Figure 5:**
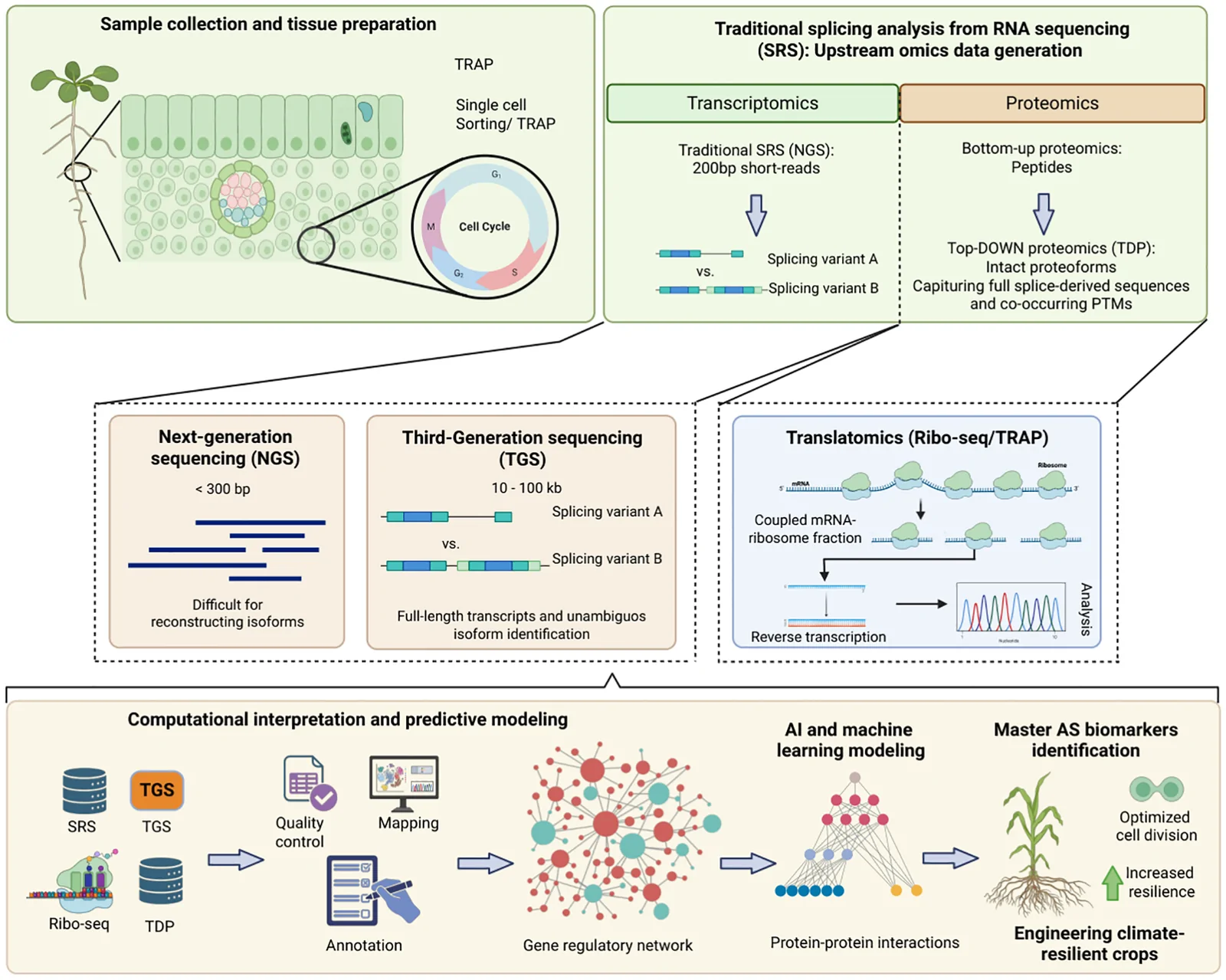
Multiomics strategies for the identification and functional characterization of alternative splicing events in plants. The scheme illustrates an integrated workflow to investigate the regulation of alternative splicing (AS) at cellular, molecular, and computational levels. Initially, plant samples are collected and processed using cell-resolution approaches, such as single-cell transcriptomics and Translating Ribosome Affinity Purification (TRAP), allowing the analysis of specific cell populations and distinct cell cycle states. Subsequently, transcriptomic and proteomic data are integrated to characterize splicing variants and their functional consequences. Conventional short-read RNA sequencing approaches, including next generation sequencing (NGS), allow the identification of AS events, whereas third-generation sequencing (TGS) enables full-length transcript reconstruction and more accurate isoform identification. Complementarily, bottom-up and top-down proteomic strategies allow the assessment of translation and proteoform diversity generated from different transcript isoforms. Translational profiling approaches, such as Ribo-seq and TRAP-seq, provide information on the association between transcripts and ribosomes, helping to distinguish potentially translated isoforms from non-translated or unstable transcripts. Created in BioRender. Araújo-lopes, B. (2026) https://BioRender.com/d8rsse3.

Altogether, emerging evidence supports the crosstalk between AS, chromatin remodeling and cell cycle progression as a regulatory hub for plant development and adaptation to the environment (Mergner and Kuster, 2022). A key challenge is how to move beyond correlation to elucidating underlying mechanisms. As noted, dual-functioning proteins CDC5, MOS4, and PRMT5 act as splice factors while coordinating transcription, chromatin remodeling, and genomic integrity, though their exact mechanistic details remain unstudied (Jia et al., 2017; Li et al., 2018; Feke et al., 2020; Guo et al., 2024; Palma et al., 2007; Lin et al., 2007). The integrated use of multi-omics workflows will unravel how plants dynamically reprogram cell cycle in response to their surroundings regarding isoform lifespan: from synthesis to phenotypic influence across unified gene regulatory networks (GRNs) (Lee and Wang, 2005; Ryan et al., 2012; Aslam et al., 2017; Zhu et al., 2023; **Figure 5**).

Since plant transcripts usually range from 1 to 3 kb (Al-Dossary et al., 2023), traditional short-read RNA sequencing (SRS) outputs are less effective for full-length splice variants reconstruction, as reads are <300bp long (Hrdlickova et al., 2016). Third-generation sequencing (TGS) technologies overcome this limitation capturing full-length, intact transcripts, with reads length between 10kb to 100kb, enabling unambiguous isoforms identification (Hu et al., 2021; **Figure 5**). However, transcription levels weakly correlate to final isoform abundance, function, and their role in the metabolism (Goldenkova-Pavlova et al., 2023). To fill this gap, translatomics approaches, such as ribosome profiling (Ribo-Seq) and translating ribosome affinity purification (TRAP), provide crucial validation, as they capture the ribosome-coupled mRNA fraction, including spliced transcripts engageds by the translational machinery during specific cell cycle phases and different tissues (Ingolia et al., 2009; Heiman et al., 2014).

To validate AS-cell cycle network dynamics, mass-spectrometry-based proteomics is an important step (Mergner and Kuster, 2022). The traditional use of bottom-up proteomics, with proteins digested into small fragments, interferes with mapping them back to the original spliced isoform (Tran et al., 2011). The recent use of top-down proteomics (TDP) solves the problem by using undigested full-length “proteoforms”, simultaneously mapping their co-occurring post-translational modifications (PTMs) (Roberts et al., 2024). Integrating single-cell and spatial proteomics ensures the identification of specific and localized splicing events (Yin et al., 2023; Guo et al., 2025), as bulk tissue sampling can obscure part of the information (**Figure 5**).

Ultimately, the large amount of high-dimensional data generated by integrated high-throughput workflows shifts the limitation from data to computational interpretation. High-quality, annotated plant genomes are the baseline for multi-omics pipelines, enabling bioinformaticians to accurately map raw reads to precise, validated, splice sites and isoforms (Jiang et al., 2017; Ji and Sadreyev, 2018; Tyagi et al., 2022). The integration of artificial intelligence and machine learning into these workflows may accelerate the discovery of dynamic protein-protein interactomes and post-translational networks (Shen et al., 2023; Nag et al., 2026). By transitioning from descriptive observations to predictive, multi-layered modeling, based on big data and collaborative repositories, scientists can employ these advanced omics to identify master AS-cell cycle biomarkers and engineer climate-resilient crops capable of optimizing cell division under shifting environmental stresses (Yan et al., 2022; Guo et al., 2025; **Figure 5**).

## References

[B1] Żabka A. Winnicki K. Polit J. T. Wróblewski M. Maszewski J. (2021). Cadmium (II)-induced oxidative stress results in replication stress and epigenetic modifications in root meristem cell nuclei of Vicia faba. Cells 10, 640. doi: 10.3390/cells10030640 33805688 PMC7999292

[B2] Ahmad S. Jose da Costa Gonzales L. Bowler-Barnett E. H. Rice D. L. Kim M. Wijerathne . (2025). The UniProt website API: facilitating programmatic access to protein knowledge. Nucleic Acids Res. 53, W547–W553. doi: 10.1093/nar/gkaf394 40331428 PMC12230682

[B3] Albaqami M. Laluk K. Reddy A. S. N. (2019). The Arabidopsis splicing regulator SR45 confers salt tolerance in a splice isoform-dependent manner. Plant Mol. Biol. 100, 379–390. doi: 10.1007/s11103-019-00864-4 30968308

[B4] Al-Dossary O. Furtado A. KharabianMasouleh A. Alsubaie B. Al-Mssallem I. Henry R. J. (2023). Long read sequencing to reveal the full complexity of a plant transcriptome by targeting both standard and long workflows. Plant Methods 19, 112–112. doi: 10.1186/s13007-023-01091-1 37865785 PMC10589961

[B5] Alhabsi A. Ling Y. Crespi M. Reddy A. S. N. Mahfouz M. (2025). Alternative splicing dynamics in plant adaptive responses to stress. Annu. Rev. Plant Biol. 76, 687–717. doi: 10.1146/annurev-arplant-083123-090055 39952682

[B6] Ali G. S. Golovkin M. Reddy A. S. N. (2003). Nuclear localization and *in vivo* dynamics of a plant-specific serine/arginine-rich protein. Plant J. 36, 883–893. doi: 10.1046/j.1365-313X.2003.01932.x 14675452

[B7] Ali G. S. Palusa S. G. Golovkin M. Prasad J. Manley J. L. Reddy A. S. N. (2007). Regulation of plant developmental processes by a novel splicing factor. PloS One 2 (5), e471. doi: 10.1371/journal.pone.0000471 17534421 PMC1868597

[B8] Aslam B. Basit M. Nisar M. A. Khurshid M. Rasool M. H. (2017). Proteomics: technologies and their applications. J. Chromatogr. Sci. 55, 182–196. doi: 10.1093/chromsci/bmw167 28087761

[B9] Barbazuk W. B. Fu Y. McGinnis K. M. (2008). Genome-wide analyses of alternative splicing in plants: opportunities and challenges. Genome Res. 18, 1381–1392. doi: 10.1101/gr.053678.106 18669480

[B10] Barrôco R. M. De Veylder L. Magyar Z. Engler G. Inzé D. Mironov V. (2003). Novel complexes of cyclin-dependent kinases and a cyclin-like protein from Arabidopsis thaliana with a function unrelated to cell division. Cell. Mol. Life. Sciences: CMLS 60, 401–412. doi: 10.1007/s000180300033 12678503 PMC11138617

[B11] Bazin J. Elvira-Matelot E. Blein T. Jauvion V. Bouteiller N. Cao J. . (2023). Synergistic action of the Arabidopsis spliceosome components PRP39a and SmD1b in promoting posttranscriptional transgene silencing. Plant Cell 35, 1917–1935. doi: 10.1093/plcell/koad091 36970782 PMC10226559

[B12] Beauchamp M. C. Alam S. S. Kumar S. Jerome-Majewska L. A. (2020). Spliceosomopathies and neurocristopathies: Two sides of the same coin? Dev. Dynamics: An. Off. Publ. Am. Assoc. Anatomists 249, 924–945. doi: 10.1002/dvdy.183 32315467

[B13] Beckel M. S. San Martín A. Sánchez S. E. Seymour D. K. de Leone M. J. Careno D. A. . (2025). Arabidopsis PRMT5 buffers pre-mRNA splicing and development against genetic variation in donor splice sites. New Phytol. 247, 1727–1741. doi: 10.1111/nph.70293 40515451

[B14] Ben-Yehuda S. Dix I. Russell C. S. McGarvey M. Beggs J. D. Kupiec M. (2000). Genetic and physical interactions between factors involved in both cell cycle progression and pre-mRNA splicing in Saccharomyces cerevisiae. Genetics 156, 1503–1517. doi: 10.1093/genetics/156.4.1503 11102353 PMC1461362

[B15] Bertram K. Agafonov D. E. Dybkov O. Haselbach D. Leelaram M. N. Will C. L. . (2017). Cryo-EM structure of a pre-catalytic human spliceosome primed for activation. Cell 170, 701–713.e11. doi: 10.1016/j.cell.2017.07.011 28781166

[B16] Bessonov S. Anokhina M. Will C. L. Urlaub H. Lührmann R. (2008). Isolation of an active step I spliceosome and composition of its RNP core. Nature 452, 846–850. doi: 10.1038/nature06842 18322460

[B17] Binns D. Dimmer E. Huntley R. Barrell D. O'Donovan C. Apweiler R. (2009). QuickGO: a web-based tool for Gene Ontology searching. Bioinformatics 25, 3045–3046. doi: 10.1093/bioinformatics/btp536 19744993 PMC2773257

[B18] Boudrez A. Beullens M. Groenen P. Van Eynde A. Vulsteke V. Jagiello I. . (2000). NIPP1-mediated interaction of protein phosphatase-1 with CDC5L, a regulator of pre-mRNA splicing and mitotic entry. J. Biol. Chem. 275, 25411–25417. doi: 10.1074/jbc.M001676200 10827081

[B19] Butt H. Bazin J. Prasad K. Awad N. Crespi M. Reddy N. . (2022). The rice serine/arginine splicing factor RS33 regulates pre-mRNA splicing during abiotic stress responses. Cells 11, 1796–1796. doi: 10.3390/cells11111796 35681491 PMC9180459

[B20] Cabezas-Fuster A. Micol-Ponce R. Fontcuberta-Cervera S. Ponce M. R. (2022). Missplicing suppressor alleles of Arabidopsis PRE-MRNA PROCESSING FACTOR 8 increase splicing fidelity by reducing the use of novel splice sites. Nucleic Acids Res. 50, 5513–5527. doi: 10.1093/nar/gkac338 35639749 PMC9177961

[B21] Carneiro A. K. Montessoro P. F. Fusaro A. F. Araújo B. G. Hemerly A. S. (2021). Plant CDKs—driving the cell cycle through climate change. Plants 10, 1804–1804. doi: 10.3390/plants10091804 34579337 PMC8468384

[B22] Carrasco-López C. Hernández-Verdeja T. Perea-Resa C. Abia D. Catalá R. Salinas J. (2017). Environment-dependent regulation of spliceosome activity by the LSM2-8 complex in Arabidopsis. Nucleic Acids Res. 45, 7416–7431. doi: 10.1093/nar/gkx375 28482101 PMC5499552

[B23] Carvalho R. F. Carvalho S. D. Duque P. (2010). The plant-specific SR45 protein negatively regulates glucose and ABA signaling during early seedling development in Arabidopsis. Plant Physiol. 154, 772–783. doi: 10.1104/pp.110.155523 20699397 PMC2949030

[B24] Cavallari N. Nibau C. Fuchs A. Dadarou D. Barta A. Doonan J. H. (2018). The cyclin‐dependent kinase G group defines a thermo‐sensitive alternative splicing circuit modulating the expression of ArabidopsisATU2AF65A. Plant J. 94, 1010–1022. doi: 10.1111/tpj.13914 29602264 PMC6032924

[B25] Chawla G. Sapra A. K. Surana U. Vijayraghavan U. (2003). Dependence of pre-mRNA introns on PRP17, a non-essential splicing factor: Implications for efficient progression through cell cycle transitions. Nucleic Acids Res. 31, 2333–2343. doi: 10.1093/nar/gkg333 12711678 PMC154219

[B26] Chen G. Chen J. Qiao Y. Shi Y. Liu W. Zeng Q. . (2018). ZNF830 mediates cancer chemoresistance through promoting homologous-recombination repair. Nucleic Acids Res. 46, 1266–1279. doi: 10.1093/nar/gkx1258 29244158 PMC5814808

[B27] Chen C. H. Chu P. C. Lee L. Lien H. W. Lin T. L. Fan C. C. . (2012). Disruption of murine mp29/Syf2/Ntc31 gene results in embryonic lethality with aberrant. 7, e33538. 10.1371/journal.pone.0033538 PMC3308990 22448250

[B28] Chen Z. H. Yu Y. P. Michalopoulos G. Nelson J. Luo J. H. (2015). The DNA replication licensing factor miniature chromosome maintenance 7 is essential for RNA splicing of epidermal growth factor receptor, c-Met, and platelet-derived growth factor receptor. J. Biol. Chem. 290, 1404–1411. doi: 10.1074/jbc.M114.622761 25425645 PMC4340387

[B29] Choi H. H. Choi H. K. Jung S. Y. Hyle J. Kim B. J. Yoon K. . (2014). CHK2 kinase promotes pre-mRNA splicing via phosphorylating CDK11(p110). Oncogene 33, 108–115. doi: 10.1038/onc.2012.535 23178491 PMC4131284

[B30] Choudhary B. Norris A. (2024). Conserved role for spliceosomal component PRPF40A in microexon splicing. RNA (New York N.Y.) 31, 43–50. doi: 10.1261/rna.080142.124 39389624 PMC11648925

[B31] Chu P. C. Wang T. Y. Lu Y. T. Chou C. K. Yang Y. C. Chang M. S. (2009). Involvement of p29 in DNA damage responses and Fanconi anemia pathway. Carcinogenesis 30 (10), 1710–1716. doi: 10.1093/carcin/bgp204 19748926

[B32] Chujo T. Scott B. (2014). Histone H3K9 and H3K27 methylation regulates fungal alkaloid biosynthesis in a fungal endophyte-plant symbiosis. Mol. Microbiol. 92, 413–434. doi: 10.1111/mmi.12567 24571357

[B33] Colwill K. Pawson T. Andrews B. Prasad J. Manley J. L. Bell J. C. . (1996). The Clk/Sty protein kinase phosphorylates SR splicing factors and regulates their intranuclear distribution. EMBO J. 15, 265–276. doi: 10.1002/j.1460-2075.1996.tb00357.x 8617202 PMC449941

[B34] Costas C. Desvoyes B. Gutierrez C. (2011). A chromatin perspective of plant cell cycle progression. Biochim. Biophys. Acta 1809, 379–387. doi: 10.1016/j.bbagrm.2011.03.005 21453801

[B35] Cruz J. O. San Martin J. A. B. Lubini G. Strini E. J. Sobral R. Pinoti V. F. . (2021). SCI1 is a direct target of AGAMOUS and WUSCHEL and is specifically expressed in the floral meristematic cells. Front. Plant Sci. 12, 642879. doi: 10.3389/fpls.2021.642879 33815449 PMC8012853

[B36] Cui X. Fan B. Scholz J. Chen Z. (2007). Roles of Arabidopsis cyclin-dependent kinase C complexes in cauliflower mosaic virus infection, plant growth, and development. Plant Cell 19, 1388–1402. doi: 10.1105/tpc.107.051375 17468259 PMC1913762

[B37] Cui D. Wang Z. Dang Q. Wang J. Qin J. Song J. . (2023). Spliceosome component Usp39 contributes to hepatic lipid homeostasis through the regulation of autophagy. Nat. Commun. 14, 7032–7032. doi: 10.1038/s41467-023-42461-6 37923718 PMC10624899

[B38] Cui P. Zhang S. Ding F. Ali S. Xiong L. (2014). Dynamic regulation of genome-wide pre-mRNA splicing and stress tolerance by the Sm-like protein LSm5 in Arabidopsis. Genome Biol. 15, R1. doi: 10.1186/gb-2014-15-1-r1 24393432 PMC4053965

[B39] Cvitkovic I. Jurica M. S. (2013). Spliceosome database: a tool for tracking components of the spliceosome. Nucleic Acids Res. 4, D132–D141. doi: 10.1093/nar/gks999 PMC3531166 23118483

[B40] Dahan O. Kupiec M. (2004). The Saccharomyces cerevisiae gene CDC40/PRP17 controls cell cycle progression through splicing of the ANC1 gene. Nucleic Acids Res. 32, 2529–2540. doi: 10.1093/nar/gkh574 15133121 PMC419462

[B41] David C. J. Manley J. L. (2010). Alternative pre-mRNA splicing regulation in cancer: Pathways and programs unhinged. Genes Dev. 24, 2343–2364. doi: 10.1101/gad.1973010 21041405 PMC2964746

[B42] de Luxán-Hernández C. Lohmann J. Tranque E. Chumova J. Binarova P. Salinas J. . (2022). MDF is a conserved splicing factor and modulates cell division and stress response in Arabidopsis. Life Sci. Alliance 6, e202201507. doi: 10.26508/lsa.202201507 36265897 PMC9585968

[B43] Deng X. Gu L. Liu C. Lu T. Lu F. Lu Z. . (2010). Arginine methylation mediated by the Arabidopsis homolog of PRMT5 is essential for proper pre-mRNA splicing. PNAS 107, 19114–19119. doi: 10.1073/pnas.1009669107 20956294 PMC2973915

[B44] Deng X. Lu T. Wang L. Gu L. Sun J. Kong X. . (2016). Recruitment of the NineTeen Complex to the activated spliceosome requires AtPRMT5. PNAS 113, 5447–5452. doi: 10.1073/pnas.1522458113 27114555 PMC4868449

[B45] DePaoli H. C. Brito M. S. Quiapim A. C. Teixeira S. P. Goldman G. H. Dornelas M. C. . (2011). Stigma/style cell cycle inhibitor 1 (SCI1), a tissue-specific cell cycle regulator that controls upper pistil development. New Phytol. 190, 882–895. doi: 10.1111/j.1469-8137.2011.03660.x 21388377

[B46] DePaoli H. C. Dornelas M. C. Goldman M. H. S. (2014). SCI1 is a component of the auxin-dependent control of cell proliferation in Arabidopsis upper pistil. Plant Science: An. Int. J. Exp. Plant Biol. 229, 122–130. doi: 10.1016/j.plantsci.2014.09.003 25443839

[B47] De Schutter K. Joubès J. Cools T. Verkest A. Corellou F. Babiychuk E. . (2007). Arabidopsis WEE1 kinase controls cell cycle arrest in response to activation of the DNA integrity checkpoint. Plant Cell 19, 211–225. doi: 10.1105/tpc.106.045047 17209125 PMC1820959

[B48] Desvoyes B. Fernández-Marcos M. Sequeira-Mendes J. Otero S. Vergara Z. Gutierrez C. (2014). Looking at plant cell cycle from the chromatin window. Front. Plant Sci. 5, 369. doi: 10.3389/fpls.2014.00369 25120553 PMC4110626

[B49] De Veylder L. Beeckman T. Inzé D. (2007). The ins and outs of the plant cell cycle. Nat. Rev. Mol. Cell Biol. 8, 655–665. doi: 10.1038/nrm2227 17643126

[B50] Dewitte W. Murray J. A. (2003). The plant cell cycle. Annu. Rev. Plant Biol. 54, 235–264. doi: 10.1146/annurev.arplant.54.031902.134836 14502991

[B51] Dominguez D. Tsai Y. H. Gomez N. Jha D. K. Davis I. Wang Z. (2016a). A high-resolution transcriptome map of cell cycle reveals novel connections between periodic genes and cancer. Cell Res. 26, 946–962. doi: 10.1038/cr.2016.84 27364684 PMC4973334

[B52] Dominguez D. Tsai Y. H. Weatheritt R. Wang Y. Blencowe B. J. Wang Z. (2016b). An extensive program of periodic alternative splicing linked to cell cycle progression. eLife 5, e10288. doi: 10.7554/eLife.10288 27015110 PMC4884079

[B53] Dong P. Tu X. Liang Z. Kang B.-H. Zhong S. (2020). Plant and animal chromatin three-dimensional organization: similar structures but different functions. J. Exp. Bot. 71, 5119–5128. doi: 10.1093/jxb/eraa220 32374833

[B54] Douglas P. Ye R. Morrice N. Britton S. Trinkle-Mulcahy L. Lees-Miller S. P. (2015). Phosphorylation of SAF-A/hnRNP-U serine 59 by polo-like kinase 1 is required for mitosis. Mol. Cell. Biol. 35, 2699–2713. doi: 10.1128/MCB.01312-14 25986610 PMC4524121

[B55] Du J.-L. Zhang S.-W. Huang H.-W. Cai T. Li L. Chen S. . (2015). The splicing factor PRP31 is involved in transcriptional gene silencing and stress response in arabidopsis. Mol. Plant 8, 1053–1068. doi: 10.1016/j.molp.2015.02.003 25684655

[B56] Dvořáčková M. Fajkus J. Mozgová I. Pečinka A. (2024). Advances in plant chromatin. Plant J. 118, 1281–1283. doi: 10.1111/tpj.16648 38814105

[B57] Elvira-Matelot E. Bardou F. Ariel F. Jauvion V. Bouteiller N. Le Masson I. . (2016). The nuclear ribonucleoprotein SmD1 interplays with splicing, RNA quality control, and posttranscriptional gene silencing in arabidopsisopen. Plant Cell 28, 426–438. doi: 10.1105/tpc.15.01045 26842463 PMC4790881

[B58] Feke A. M. Hong J. Liu W. Gendron J. M. (2020). A decoy library uncovers U-box E3 ubiquitin ligases that regulate flowering time in arabidopsis. Genetics 215, 699–712. doi: 10.1534/genetics.120.303199 32434795 PMC7337086

[B59] Ganie S. A. Reddy A. S. N. (2021). Stress-induced changes in alternative splicing landscape in rice: functional significance of splice isoforms in stress tolerance. Biology 10, 309. doi: 10.3390/biology10040309 33917813 PMC8068108

[B60] Gill S. S. Tuteja N. (2010). Reactive oxygen species and antioxidant machinery in abiotic stress tolerance in crop plants. Plant Physiol. Biochem. 48, 909–930. doi: 10.1016/j.plaphy.2010.08.016 20870416

[B61] Godoy Herz M. A. Kornblihtt A. R. (2019). Alternative splicing and transcription elongation in plants. Front. Plant Sci. 10, 309. doi: 10.3389/fpls.2019.00309 30972082 PMC6443983

[B62] Godwin J. Farrona S. (2020). Plant epigenetic stress memory induced by drought: a physiological and molecular perspective. Methods Mol. Biol. 2093, 243–259. doi: 10.1007/978-1-0716-0179-2_17 32088901

[B63] Goldenkova-Pavlova I. V. Mustafaev O. N. Fridman V. A. Deyneko I. V. Tyurin A. A. (2023). On the way to translatomic mapping, a state-of-the-art. Russ. J. Plant Physiol. 70. doi: 10.1134/S1021443723603026

[B64] Golovkin M. Reddy A. S. (1996). Structure and expression of a plant U1 snRNP 70K gene: alternative splicing of U1 snRNP 70K pre-mRNAs produces two different transcripts. Plant Cell 8, 1421–1435. doi: 10.1105/tpc.8.8.1421 8776903 PMC161266

[B65] Golovkin M. Reddy A. S. (1998). The plant U1 small nuclear ribonucleoprotein particle 70K protein interacts with two novel serine/arginine-rich proteins. Plant Cell 10, 1637–1648. doi: 10.1105/tpc.10.10.1637 9761791 PMC143944

[B66] Golovkin M. Reddy A. S. (1999). An SC35-like protein and a novel serine/arginine-rich protein interact with arabidopsis U1-70K protein. J. Biol. Chem. 274, 36428–36438. doi: 10.1074/jbc.274.51.36428 10593939

[B67] Golovkin M. Reddy A. S. (2003). Expression of U1 small nuclear ribonucleoprotein 70K antisense transcript using APETALA3 promoter suppresses the development of sepals and petals. Plant Physiol. 132, 1884–1891. doi: 10.1104/pp.103.023192 12913145 PMC181274

[B68] Gonzalez N. Vanhaeren H. Inzé D. (2012). Leaf size control: complex coordination of cell division and expansion. Trends Plant Sci. 17, 332–340. doi: 10.1016/j.tplants.2012.02.003 22401845

[B69] Graveley B. R. (2000). Sorting out the complexity of SR protein functions. RNA 6, 1197–1210. doi: 10.1017/S1355838200000960 10999598 PMC1369994

[B70] Grote M. Wolf E. Will C. L. Lemm I. Agafonov D. E. Schomburg A. . (2010). Molecular architecture of the human Prp19/CDC5L complex. Mol. Cell. Biol. 30, 2105–2119. doi: 10.1128/MCB.01505-09 20176811 PMC2863591

[B71] Gu J. Xia Z. Luo Y. Jiang X. Qian B. Xie H. . (2018). Spliceosomal protein U1A is involved in alternative splicing and salt stress tolerance in Arabidopsis thaliana. Nucleic Acids Res. 46, 1777–1792. doi: 10.1093/nar/gkx1229 29228330 PMC5829640

[B72] Guo T. Steen J. A. Mann M. (2025). Mass-spectrometry-based proteomics: from single cells to clinical applications. Nature 638, 901–911. doi: 10.1038/s41586-025-08584-0 40011722

[B73] Guo X. Wang T. Jiang L. Qi H. Zhang Z. (2023). PlaASDB: a comprehensive database of plant alternative splicing events in response to stress. BMC Plant Biol. 23, 25. doi: 10.1186/s12870-023-04234-7 37106367 PMC10134664

[B74] Guo J. Yang L. Huang J. Liu X. Qiu X. Tao T. . (2014). Knocking down the expression of SYF2 inhibits the proliferation of glioma cells. Med. Oncol. (Northwood London England) 31, 101. doi: 10.1007/s12032-014-0101-x 24985881

[B75] Guo X. Zhang X. Jiang S. Qiao X. Meng B. Wang X. . (2024). E3 ligases MAC3A and MAC3B ubiquitinate UBIQUITIN-SPECIFIC PROTEASE14 to regulate organ size in arabidopsis. Plant Physiol. 194, 684–697. doi: 10.1093/plphys/kiad559 37850874 PMC10828200

[B76] He X. Zhang P. (2015). Serine/arginine-rich splicing factor 3 (SRSF3) regulates homologous recombination-mediated DNA repair. Mol. Cancer 14, 158. doi: 10.1186/s12943-015-0422-1 26282282 PMC4539922

[B77] Heiman M. Kulicke R. Fenster R. J. Greengard P. Heintz N. (2014). Cell type-specific mRNA purification by translating ribosome affinity purification (TRAP). Nat. Protoc. 9, 1282–1291. doi: 10.1038/nprot.2014.085 24810037 PMC4102313

[B78] Hernando C. E. Sanchez S. E. Mancini E. Yanovsky M. J. (2015). Genome wide comparative analysis of the effects of PRMT5 and PRMT4/CARM1 arginine methyltransferases on the Arabidopsis thaliana transcriptome. BMC Genomics 16, 192. doi: 10.1186/s12864-015-1399-2 25880665 PMC4381356

[B79] Hluchý M. Gajdušková P. Ruiz de los Mozos I. Rájecký M. Kluge M. Berger B. T. . (2022). CDK11 regulates pre-mRNA splicing by phosphorylation of SF3B1. Nature 609, 829–834. doi: 10.1038/s41586-022-05204-z 36104565

[B80] Hong Y. Gao Y. Pang J. Shi H. Li T. Meng H. . (2023). The Sm core protein SmEb regulates salt stress responses through maintaining proper splicing of RCD1 pre‐mRNA in Arabidopsis. J. Integr. Plant Biol. 65, 1383–1393. doi: 10.1111/jipb.13457 36661041

[B81] Hrdlickova R. Toloue M. Tian B . (2016). RNA-Seq methods for transcriptome analysis. Wiley interdisciplinary reviews. RNA, 8(1). doi: 10.1002/wrna.1364 PMC5717752 27198714

[B82] Hong Y. Yao J. Shi H. Chen Y. Zhu J.-K. Wang Z. (2021). The Arabidopsis spliceosomal protein SmEb modulates ABA responses by maintaining proper alternative splicing of HAB1. Stress Biol. 1, 8. doi: 10.1007/s44154-021-00006-1 37676319 PMC10441929

[B83] Hu T. Chitnis N. Monos D. Dinh A. (2021). Next-generation sequencing technologies: an overview. Hum. Immunol. 82, 801–811. doi: 10.1016/j.humimm.2021.02.012 33745759

[B84] Hu D. Mayeda A. Trembley J. H. Lahti J. M. Kidd V. J. (2003). CDK11 complexes promote pre-mRNA splicing. J. Biol. Chem. 278, 8623–8629. doi: 10.1074/jbc.M210057200 12501247

[B85] Hu D. Thériault B. L. Talebian V. Hoffer L. Owen J. Lim J. . (2025). CDC40 suppression induces CDCA5 splicing defects and anti-proliferative effects in lung cancer cells. Sci. Rep. 15, 315. doi: 10.1038/s41598-024-83337-z 39747150 PMC11696760

[B86] Huang C.-F. Miki D. Tang K. Zhou H.-R. Zheng Z. Chen W. . (2013a). A pre-mRNA-splicing factor is required for RNA-directed DNA methylation in arabidopsis. PloS Genet. 9, e1003779. doi: 10.1371/journal.pgen.1003779 24068953 PMC3772050

[B87] Huang X. Y. Niu J. Sun M. X. Zhu J. Gao J. F. Yang J. . (2013b). CYCLIN-DEPENDENT KINASE G1 is associated with the spliceosome to regulate CALLOSE SYNTHASE5 splicing and pollen wall formation in arabidopsis. Plant Cell 25, 637–648. doi: 10.1105/tpc.112.107896 23404887 PMC3608783

[B88] Huang Y. Wu J. Li X. Wang J. Ma M. Jiang W. . (2025). The Arabidopsis histone methylation reader MRG2 interacts with eIF4A3 to regulate alternative splicing and circadian rhythms. Plant Cell 37 (8). doi: 10.1093/plcell/koaf209 40825069

[B89] Huang X. Liu Y. Cai Y. Long X. Xu E. Zhong X. . (2026). Key divisions and cell specification during embryo pattern formation require SMU1-mediated splicing of CAK genes in Arabidopsis. Journal of genetics and genomics. 53(4), 704–718. doi: 10.1016/j.jgg.2025.10.008 41241148

[B90] Huo Z. Zhai S. Weng Y. Qian H. Tang X. Shi Y. . (2019). PRPF40A as a potential diagnostic and prognostic marker is upregulated in pancreatic cancer tissues and cell lines: an integrated bioinformatics data analysis. OncoTargets Ther. 12, 5037–5051. doi: 10.2147/OTT.S206039 31303762 PMC6610298

[B91] Iglesias F. M. Cerdán P. D. (2016). Maintaining epigenetic inheritance during DNA replication in plants. Front. Plant Sci. 7, 38. doi: 10.3389/fpls.2016.00038 26870059 PMC4735446

[B92] Iida K. Go M. (2006). Survey of conserved alternative splicing events of mRNAs encoding SR proteins in land plants. Mol. Biol. Evol. 23, 1085–1094. doi: 10.1093/molbev/msj118 16520337

[B93] Ingolia N. T. Ghaemmaghami S. Newman J. R. Weissman J. S. (2009). Genome-wide analysis *in vivo* of translation with nucleotide resolution using ribosome profiling. Sci. (New York N.Y.) 324, 218–228. doi: 10.1126/science.1168978 19213877 PMC2746483

[B94] Inzé D. De Veylder L. (2006). Cell cycle regulation in plant development. Annu. Rev. Genet. 40, 77–105. doi: 10.1146/annurev.genet.40.110405.090431 17094738

[B95] Isshiki M. Tsumoto A. Shimamoto K. (2006). The serine/arginine-rich protein family in rice plays important roles in constitutive and alternative splicing of pre-mRNA. Plant Cell 18, 146–158. doi: 10.1105/tpc.105.037069 16339852 PMC1323490

[B96] Jabre I. Chaudhary S. Wilson C. M. Staiger D. Syed N. (2022). Stochastic variation in DNA methylation modulates nucleosome occupancy and alternative splicing in Arabidopsis thaliana. Plants 11, 1105. doi: 10.3390/plants11091105 35567106 PMC9101026

[B97] Jabre I. Reddy A. S. N. Kalyna M. Chaudhary S. Khokhar W. Byrne L. J. . (2019). Does co-transcriptional regulation of alternative splicing mediate plant stress responses? Nucleic Acids Res. 47, 2716–2726. doi: 10.1093/nar/gkz121 30793202 PMC6451118

[B98] Ji F. Sadreyev R. I. (2018). RNA-seq: basic bioinformatics analysis. Curr. Protoc. Mol. Biol. 124, e68. doi: 10.1002/cpmb.68 30222249 PMC6168365

[B99] Jia R. Ajiro M. Yu L. McCoy P. J. Zheng Z. M. (2019). Oncogenic splicing factor SRSF3 regulates ILF3 alternative splicing to promote cancer cell proliferation and transformation. RNA (New York N.Y.) 25, 630–644. doi: 10.1261/rna.068619.118 30796096 PMC6467003

[B100] Jia R. Li C. McCoy J. P. Deng C. X. Zheng Z. M. (2010). SRp20 is a proto-oncogene critical for cell proliferation and tumor induction and maintenance. Int. J. Biol. Sci. 6, 806–826. doi: 10.7150/ijbs.6.806 21179588 PMC3005347

[B101] Jia J. Long Y. Zhang H. Li Z. Liu Z. Zhao Y. . (2020). Post-transcriptional splicing of nascent RNA contributes to widespread intron retention in plants. Nat. Plants 6, 780–788. doi: 10.1038/s41477-020-0688-1 32541953

[B102] Jia T. Zhang B. You C. Zhang Y. Zeng L. Li S. . (2017). The arabidopsis MOS4-associated complex promotes microRNA biogenesis and precursor messenger RNA splicing. Plant Cell 29, 2626–2643. doi: 10.1105/tpc.17.00370 28947490 PMC5774577

[B103] Jiang X. Hall A. B. Biedler J. K. Tu Z. (2017). Single molecule RNA sequencing uncovers trans-splicing and improves annotations in Anopheles stephensi. Insect Mol. Biol. 26, 298–307. doi: 10.1111/imb.12294 28181326 PMC5718059

[B104] Jiang S. Wei J. Li N. Wang Z. Zhang Y. Xu R. . (2022). The UBP14-CDKB1;1-CDKG2 cascade controls endoreduplication and cell growth in arabidopsis. Plant Cell 34, 1308–1325. doi: 10.1093/plcell/koac002 34999895 PMC8972217

[B105] Jin Y. Ivanov M. Dittrich A. N. Nelson A. D. Marquardt S. (2023). LncRNA FLAIL affects alternative splicing and represses flowering in Arabidopsis. EMBO J. 42, e110921. doi: 10.15252/embj.2022110921 37051749 PMC10233378

[B106] Jokoji G. Maeda S. Oishi K. Ijuin T. Nakajima M. Tawaratsumida H. . (2021). CDC5L promotes early chondrocyte differentiation and proliferation by modulating pre-mRNA splicing of SOX9, COL2A1, and WEE1. J. Biol. Chem. 297, 100994. doi: 10.1016/j.jbc.2021.100994 34298017 PMC8363834

[B107] Kalsotra A. Cooper T. A. (2011). Functional consequences of developmentally regulated alternative splicing. Nat. Rev. Genet. 12, 715–729. doi: 10.1038/nrg3052 21921927 PMC3321218

[B108] Kalyna M. Barta A. (2004). A plethora of plant serine/arginine-rich proteins: redundancy or evolution of novel gene functions? Biochem. Soc Trans. 32, 561–564. doi: 10.1042/bst0320561 15270675 PMC5362061

[B109] Kaplan Y. Kupiec M. (2007). A role for the yeast cell cycle/splicing factor Cdc40 in the G1/S transition. Curr. Genet. 51, 123–140. doi: 10.1007/s00294-006-0113-y 17171376

[B110] Karamysheva Z. Díaz-Martínez L. A. Warrington R. Yu H. (2015). Graded requirement for the spliceosome in cell cycle progression. Cell. Cycle 14, 1873–1883. doi: 10.1080/15384101.2015.1039209 25892155 PMC4614359

[B111] Khusial P. R. Vaidya K. Zieve G. W. (2005). The symmetrical dimethylarginine post-translational modification of the SmD3 protein is not required for snRNP assembly and nuclear transport. Biochem. Biophys. Res. Commun. 337, 1119–1124. doi: 10.1016/j.bbrc.2005.09.161 16236255

[B112] Kim J.-H. (2019). Chromatin remodeling and epigenetic regulation in plant DNA damage repair. Int. J. Mol. Sci. 20, 4093. doi: 10.3390/ijms20174093 31443358 PMC6747262

[B113] Kim G.-D. Cho Y.-H. Lee B.-H. Yoo S.-D. (2017). STABILIZED1 modulates pre-mRNA splicing for thermotolerance. Plant Physiol. 173, 2370–2382. doi: 10.1104/pp.16.01928 28223317 PMC5373063

[B114] Kim M. K. Nikodem V. M. (1999). hnRNP U inhibits carboxy-terminal domain phosphorylation by TFIIH and represses RNA polymerase II elongation. Mol. Cell. Biol. 19, 6833–6844. doi: 10.1128/MCB.19.10.6833 10490622 PMC84680

[B115] Kitsios G. Alexiou K. G. Bush M. Shaw P. Doonan J. H. (2008). A cyclin-dependent protein kinase, CDKC2, colocalizes with and modulates the distribution of spliceosomal components in Arabidopsis. Plant J. 54, 220–235. doi: 10.1111/j.1365-313X.2008.03414.x 18208522

[B116] Komaki S. Sugimoto K. (2012). Control of the plant cell cycle by developmental and environmental cues. Plant Cell Physiol. 53, 953–964. doi: 10.1093/pcp/pcs070 22555815

[B117] Kornblihtt A. R. Schor I. E. Alló M. Dujardin G. Petrillo E. Muñoz M. J. (2013). Alternative splicing: a pivotal step between eukaryotic transcription and translation. Nat. Rev. Mol. Cell Biol. 14, 153–165. doi: 10.1038/nrm3525 23385723

[B118] Kambach C. Walke S. Young R. Avis J. M. de la Fortelle E. Raker V. A. . (1999). Crystal structures of two Sm protein complexes and their implications for the assembly of the spliceosomal snRNPs. Cell, 96(3), 375–387. doi: 10.1016/s0092-8674(00)80550-4 10025403

[B119] Kuraoka I. Ito S. Wada T. Hayashida M. Lee L. Saijo M. . (2008). Isolation of XAB2 complex involved in pre-mRNA splicing, transcription, and transcription-coupled repair. J. Biol. Chem. 283, 940–950. doi: 10.1074/jbc.M706647200 17981804

[B120] Kurokawa K. Akaike Y. Masuda K. Kuwano Y. Nishida K. Yamagishi N. . (2014). Downregulation of serine/arginine-rich splicing factor 3 induces G1 cell cycle arrest and apoptosis in colon cancer cells. Oncogene 33, 1407–1417. doi: 10.1038/onc.2013.86 23503458

[B121] Laloum T. Carvalho S. D. Martín G. Richardson D. N. Cruz T. M. D. Carvalho R. F. . (2023). The SCL30a SR protein regulates ABA-dependent seed traits and germination under stress. Plant Cell Environ. 46, 2112–2127. doi: 10.1111/pce.14593 37098235

[B122] Laloum T. Martín G. Duque P. (2018). Alternative splicing control of abiotic stress responses. Trends Plant Sci. 23, 140–150. doi: 10.1016/j.tplants.2017.09.019 29074233

[B123] Lee B. Kapoor A. Zhu J. Zhu J.-K. (2006). STABILIZED1, a stress-upregulated nuclear protein, is required for pre-mRNA splicing, mRNA turnover, and stress tolerance in Arabidopsis. Plant Cell 18, 1736–1749. doi: 10.1105/tpc.106.042184 16751345 PMC1488911

[B124] Lee C. Wang Q. (2005). Bioinformatics analysis of alternative splicing. Briefings Bioinf. 6, 23–33. doi: 10.1093/bib/6.1.23 15826354

[B125] Li Y. Guo Q. Liu P. Huang J. Zhang S. Yang G. . (2021a). Dual roles of the serine/arginine-rich splicing factor SR45a in promoting and interacting with nuclear cap-binding complex to modulate the salt-stress response in Arabidopsis. New Phytol. 230, 641–655. doi: 10.1111/nph.17175 33421141

[B126] Li S. Liu K. Zhou B. Li M. Zhang S. Zeng L. . (2018). MAC3A and MAC3B, two core subunits of the MOS4-associated complex, positively influence miRNA biogenesis. Plant Cell 30, 481–494. doi: 10.1105/tpc.17.00953 29437988 PMC5868694

[B127] Li X. Wang X. Zhao J. Wang J. Wu J. (2021b). PRMT5 promotes colorectal cancer growth by interaction with MCM7. J. Cell. Mol. Med. 25, 3537–3547. doi: 10.1111/jcmm.16436 33675123 PMC8034445

[B128] Li X. Yuan J. Song C. Lei Y. Xu J. Zhang G. . (2021c). Deubiquitinase USP39 and E3 ligase TRIM26 balance the level of ZEB1 ubiquitination and thereby determine the progression of hepatocellular carcinoma. Cell death and differentiation, 28(8), 2315–2332. doi: 10.1038/s41418-021-00754-7 33649471 PMC8329202

[B129] Li Q. Zhao Y. Yue M. Xue Y. Bao S. (2016). The protein arginine methylase 5 (PRMT5/SKB1) gene is required for the maintenance of root stem cells in response to DNA damage. J. Genet. Genomics 43, 187–197. doi: 10.1016/j.jgg.2016.02.007 27090604

[B130] Li Y. Zhu Q. Zhou S. Chen J. Du A. Qin C. (2023). Combined bulk RNA and single-cell RNA analyses reveal TXNL4A as a new biomarker for hepatocellular carcinoma. Front. Oncol. 13, 1202732. doi: 10.3389/fonc.2023.1202732 37305572 PMC10248245

[B131] Liang Y. Fan Y. Liu Y. Fan H. (2021). HNRNPU promotes the progression of hepatocellular carcinoma by enhancing CDK2 transcription. Exp. Cell. Res. 409, 112898. doi: 10.1016/j.yexcr.2021.112898 34737140

[B132] Lin Z. Yin K. Zhu D. Chen Z. Gu H. Qu L. J. (2007). AtCDC5 regulates the G2 to M transition of the cell cycle and is critical for the function of Arabidopsis shoot apical meristem. Cell Res. 17, 815–828. doi: 10.1038/cr.2007.71 17768399

[B133] Liu X. X. Guo Q. H. Xu W. B. Liu P. Yan K. (2022). Rapid regulation of alternative splicing in response to environmental stresses. Front. Plant Sci. 13, 832177. doi: 10.3389/fpls.2022.832177 35310672 PMC8931528

[B134] Liu H. Ma X. Han H. Hao Y. Zhang X. (2016). AtPRMT5 regulates shoot regeneration through mediating histone H4R3 dimethylation on KRPs and pre-mRNA splicing of RKP in Arabidopsis. Mol. Plant 9, 1634–1646. doi: 10.1016/j.molp.2016.10.010 27780782

[B135] Liu S. Rauhut R. Vornlocher H. P. Lührmann R. (2006). The network of protein-protein interactions within the human U4/U6.U5 tri-snRNP. RNA (New York N.Y.) 12, 1418–1430. doi: 10.1261/rna.55406 16723661 PMC1484429

[B136] Liu G. Wang H. Gao H. Yu S. Liu C. Wang Y. . (2025). Alternative splicing of functional genes in plant growth, development, and stress responses. Int. J. Mol. Sci. 26, 5864. doi: 10.3390/ijms26125864 40565329 PMC12193667

[B137] Lucibelli F. Valoroso M. C. Aceto S. (2022). Plant DNA methylation: an epigenetic mark in development, environmental interactions, and evolution. Int. J. Mol. Sci. 23, 8299. doi: 10.3390/ijms23158299 35955429 PMC9368846

[B138] Luco R. F. Allo M. Schor I. E. Kornblihtt A. R. Misteli T. (2011). Epigenetics in alternative pre-mRNA splicing. Cell 144, 16–26. doi: 10.1016/j.cell.2010.11.056 21215366 PMC3038581

[B139] Ma J. Li S. Wang T. Tao Z. Huang S. Lin N. . (2025). Cooperative condensation of RNA-DIRECTED DNA METHYLATION 16 splicing isoforms enhances heat tolerance in Arabidopsis. Nat. Commun. 16, 433–433. doi: 10.1038/s41467-025-55850-w 39762263 PMC11704304

[B140] Ma N. Matsunaga S. Morimoto A. Sakashita G. Urano T. Uchiyama S. . (2011). The nuclear scaffold protein SAF-A is required for kinetochore-microtubule attachment and contributes to the targeting of Aurora-A to mitotic spindles. J. Cell Sci. 124, 394–404. doi: 10.1242/jcs.063347 21242313

[B141] Ma X. Qiao Z. Chen D. Yang W. Zhou R. Zhang W. . (2015). CYCLIN-DEPENDENT KINASE G2 regulates salinity stress response and salt mediated flowering in Arabidopsis thaliana. Plant molecular biology, 88(3), 287–299. doi: 10.1007/s11103-015-0324-z 25948280

[B142] Mandadi K. K. Petrillo E. Dubrovina A. S. Kiselev K. V. (2023). Editorial: Regulation of alternative splicing in plant stress responses. Front. Plant Sci. 13, 1120961. doi: 10.3389/fpls.2022.1120961 36733599 PMC9888408

[B143] Mao S. Li Y. Lu Z. Che Y. Huang J. Lei Y. . (2019). PHD finger protein 5A promoted lung adenocarcinoma progression via alternative splicing. Cancer Med. 8, 2429–2441. doi: 10.1002/cam4.2115 30932358 PMC6536992

[B144] Maréchal A. Li J.-M. Ji X. Wu C.-S. Yazinski S. A. Nguyen H. . (2014). PRP19 transforms into a sensor of RPA-ssDNA after DNA damage and drives ATR activation via a ubiquitin-mediated circuitry. Mol. Cell 53, 235–246. doi: 10.1016/j.molcel.2013.11.002 24332808 PMC3946837

[B145] Martín G. Márquez Y. Mantica F. Duque P. Irimia M. (2021). Alternative splicing landscapes in Arabidopsis thaliana across tissues and stress conditions highlight major functional differences with animals. Genome Biol. 22, 35. doi: 10.1186/s13059-020-02258-y 33446251 PMC7807721

[B146] Mejias J. Bazin J. Truong N. M. Chen Y. Marteu N. Bouteiller N. . (2021). The root-knot nematode effector MiEFF18 interacts with the plant core spliceosomal protein SmD1 required for giant cell formation. New Phytol. 229, 3408–3423. doi: 10.1111/nph.17089 33206370

[B147] Mejias J. Chen Y. Bazin J. Truong N. M. Mulet K. Noureddine Y. . (2022). Silencing the conserved small nuclear ribonucleoprotein SmD1 target gene alters susceptibility to root-knot nematodes in plants. Plant Physiol. 189, 1741–1756. doi: 10.1093/plphys/kiac155 35385078 PMC9237699

[B148] Menges M. de Jager S. M. Gruissem W. Murray J. A. (2005). Global analysis of the core cell cycle regulators of Arabidopsis identifies novel genes, reveals multiple and highly specific profiles of expression and provides a coherent model for plant cell cycle control. Plant Journal: For. Cell. Mol. Biol. 41, 546–566. doi: 10.1111/j.1365-313X.2004.02319.x 15686519

[B149] Mergner J. Kuster B. (2022). Plant proteome dynamics. Annu. Rev. Plant Biol. 73, 67–92. doi: 10.1146/annurev-arplant-102620-031308 35138880

[B150] Mu R. Wang Y. B. Wu M. Yang Y. Song W. Li T. . (2014). Depletion of pre-mRNA splicing factor Cdc5L inhibits mitotic progression and triggers mitotic catastrophe. Cell Death Dis. 5, e1151. doi: 10.1038/cddis.2014.117 24675469 PMC3973201

[B151] Muhammad S. Xu X. Zhou W. Wu L. (2023). Alternative splicing: an efficient regulatory approach towards plant developmental plasticity. Wiley Interdiscip. Rev. RNA 14, e1758. doi: 10.1002/wrna.1758 35983878

[B152] Naftelberg S. Schor I. E. Ast G. Kornblihtt A. R. (2015). Regulation of alternative splicing through coupling with transcription and chromatin structure. Annu. Rev. Biochem. 84, 165–198. doi: 10.1146/annurev-biochem-060614-034242 26034889

[B153] Nag A. Sil R. Hassan M. M. Ritu T. H. Das B. Roy N. . (2026). Exploring the power of machine learning in analysing protein-protein sequences. IET Syst. Biol. 20, e70066. doi: 10.1049/syb2.70066 42018649 PMC13102167

[B154] Ner-Gaon H. Halachmi R. Savaldi-Goldstein S. Rubin E. Ophir R. Fluhr R. (2004). Intron retention is a major phenomenon in alternative splicing in Arabidopsis. Plant Journal: For. Cell. Mol. Biol. 39, 877–885. doi: 10.1111/j.1365-313X.2004.02172.x 15341630

[B155] Nibau C. Gallemí M. Dadarou D. Doonan J. H. Cavallari N. (2020a). Thermo-sensitive alternative splicing of FLOWERING LOCUS M is modulated by cyclin-dependent kinase G2. Front. Plant Sci. 10. doi: 10.3389/fpls.2019.01680 32038671 PMC6987439

[B156] Nibau C. Lloyd A. Dadarou D. Betekhtin A. Tsilimigka F. Phillips D. W. . (2020b). CDKG1 is required for meiotic and somatic recombination intermediate processing in Arabidopsis[CC-BY. Plant Cell 32, 1308–1322. doi: 10.1105/TPC.19.00942 32047050 PMC7145484

[B157] Nimeth B. A. Riegler S. Kalyna M. (2020). Alternative splicing and DNA damage response in plants. Front. Plant Sci. 11. doi: 10.3389/fpls.2020.00091 32140165 PMC7042379

[B158] Nisa M. U. Huang Y. Benhamed M. Raynaud C. (2019). The plant DNA damage response: Signaling pathways leading to growth inhibition and putative role in response to stress conditions. Front. Plant Sci. 10, 653. doi: 10.3389/fpls.2019.00653 31164899 PMC6534066

[B159] Nozawa R. S. Boteva L. Soares D. C. Naughton C. Dun A. R. Buckle A. . (2017). SAF-A regulates interphase chromosome structure through oligomerization with chromatin-associated RNAs. Cell 169, 1214–1227.e18. doi: 10.1016/j.cell.2017.05.029 28622508 PMC5473940

[B160] Ogita N. Okushima Y. Tokizawa M. Yamamoto Y. Y. Tanaka M. Seki M. . (2018). Identifying the target genes of SUPPRESSOR OF GAMMA RESPONSE 1, a master transcription factor controlling DNA damage response in Arabidopsis. Plant J. 94, 439–453. doi: 10.1111/tpj.13866 29430765

[B161] Okamoto M. Matsui A. Tanaka M. Morosawa T. Ishida J. Iida K. . (2016). Sm-like protein-mediated RNA metabolism is required for heat stress tolerance in Arabidopsis. Front. Plant Sci. 7. doi: 10.3389/fpls.2016.01079 27493656 PMC4954817

[B162] Oltean S. Bates D. O. (2014). Hallmarks of alternative splicing in cancer. Oncogene 33, 5311–5318. doi: 10.1038/onc.2013.533 24336324

[B163] Pajoro A. Severing E. Angenent G. C. Immink R. G. H. (2017). Histone H3 lysine 36 methylation affects temperature-induced alternative splicing and flowering in plants. Genome Biol. 18, 102. doi: 10.1186/s13059-017-1235-x 28566089 PMC5452352

[B164] Palma K. Zhao Q. Yu T. C. Bi D. Monaghan J. Cheng W. . (2007). Regulation of plant innate immunity by three proteins in a complex conserved across the plant and animal kingdoms. Genes Dev. 21, 1484–1493. doi: 10.1101/gad.1559607 17575050 PMC1891426

[B165] Pan Q. Shai O. Lee L. J. Frey B. J. Blencowe B. J. (2008). Deep surveying of alternative splicing complexity in the human transcriptome by high-throughput sequencing. Nat. Genet. 40, 1413–1415. doi: 10.1038/ng.259 18978789

[B166] Park H. Y. Lee H. T. Lee J. H. Kim J. K. (2019). Arabidopsis U2AF65 regulates flowering time and the growth of pollen tubes. Front. Plant Sci. 10, 569. doi: 10.3389/fpls.2019.00569 31130976 PMC6510283

[B167] Park B. Y. Tachi-Duprat M. Ihewulezi C. Devotta A. Saint-Jeannet J. P. (2022). The core splicing factors EFTUD2, SNRPB and TXNL4A are essential for neural crest and craniofacial development. J. Dev. Biol. 10, 29. doi: 10.3390/jdb10030029 35893124 PMC9326569

[B168] Pei Y. Niu L. Lu F. Liu C. Zhai J. Kong X. . (2007). Mutations in the type II protein arginine methyltransferase AtPRMT5 result in pleiotropic developmental defects in Arabidopsis. Plant Physiol. 144, 1913–1923. doi: 10.1104/pp.107.099531 17573539 PMC1949897

[B169] Petasny M. Bentata M. Pawellek A. Baker M. Kay G. Salton M. (2021). Splicing to keep cycling: The importance of pre-mRNA splicing during the cell cycle. Trends Genet. TIG 37, 266–278. doi: 10.1016/j.tig.2020.08.013 32950269

[B170] Pinoti V. F. Ferreira P. B. Strini E. J. Lubini G. Thomé V. Cruz J. . (2024). SCI1, a flower regulator of cell proliferation, and its partners NtCDKG2 and NtRH35 interact with the splicing machinery. J. Exp. Bot. 75, 6312–6330. doi: 10.1093/jxb/erae337 39113673

[B171] Polprasert C. Schulze I. Sekeres M. A. Makishima H. Przychodzen B. Hosono N. . (2015). Inherited and somatic defects in DDX41 in myeloid neoplasms. Cancer Cell. 27, 658–670. doi: 10.1016/j.ccell.2015.03.017 25920683 PMC8713504

[B172] Qadir M. Kaur N. Rahman F. U. Nabi F. Ahmed Z. F. R. Wu J. (2026). Epigenetic modifications in plant abiotic stress adaptation: Towards climate-resilient and sustainable crop improvement. Front. Plant Sci. 17, 1738299. doi: 10.3389/fpls.2026.1738299 41756647 PMC12932597

[B173] Qi F. Zhang F. (2020). Cell cycle regulation in the plant response to stress. Front. Plant Sci. 10. doi: 10.3389/fpls.2019.01765 32082337 PMC7002440

[B174] Reddy A. S. (2007). Alternative splicing of pre-messenger RNAs in plants in the genomic era. Annu. Rev. Plant Biol. 58, 267–294. doi: 10.1146/annurev.arplant.58.032806.103754 17222076

[B175] Reddy A. S. Marquez Y. Kalyna M. Barta A . (2013). Complexity of the alternative splicing landscape in plants. The Plant cell, 25(10), 3657–3683. doi: 10.1105/tpc.113.117523 24179125 PMC3877793

[B176] Rigo R. Bazin J. Romero-Barrios N. Moison M. Lucero L. Christ A. . (2020). The Arabidopsis lncRNA ASCO modulates the transcriptome through interaction with splicing factors. EMBO Rep. 21, e48977. doi: 10.15252/embr.201948977 32285620 PMC7202219

[B177] Roberts D. S. Loo J. A. Tsybin Y. O. Liu X. Wu S. Chamot-Rooke J. . (2024). Top-down proteomics. Nat. Rev. Methods Primers 4, 38. doi: 10.1038/s43586-024-00318-2 39006170 PMC11242913

[B178] Ryan M. C. Cleland J. Kim R. Wong W. C. Weinstein J. N. (2012). SpliceSeq: A resource for analysis and visualization of RNA-Seq data on alternative splicing and its functional impacts. Bioinf. (Oxford England) 28, 2385–2387. doi: 10.1093/bioinformatics/bts452 22820202 PMC3436850

[B179] Sablowski R. Gutierrez C. (2022). Cycling in a crowd: Coordination of plant cell division, growth, and cell fate. Plant Cell 34, 193–208. doi: 10.1093/plcell/koab222 34498091 PMC8774096

[B180] Saldivar J. C. Cortez D. Cimprich K. A. (2017). The essential kinase ATR: Ensuring faithful duplication of a challenging genome. Nat. Rev. Mol. Cell Biol. 18, 622–636. doi: 10.1038/nrm.2017.67 28811666 PMC5796526

[B181] Sanchez M. Caro E. Desvoyes B. Ramirez-Parra E. Gutierrez C. (2008). Chromatin dynamics during the plant cell cycle. Semin. Cell. Dev. Biol. 19, 537–546. doi: 10.1016/j.semcdb.2008.07.014 18707013

[B182] Sanchez S. E. Petrillo E. Beckwith E. J. Zhang X. Rugnone M. L. Hernando C. E. . (2010). A methyl transferase links the circadian clock to the regulation of alternative splicing. Nature 468, 112–116. doi: 10.1038/nature09470 20962777

[B183] Sánchez-García A. B. Aguilera V. Micol-Ponce R. Jover-Gil S. Ponce M. R. (2015). Arabidopsis MAS2, an essential gene that encodes a homolog of animal NF-κ B activating protein, is involved in 45S ribosomal DNA silencing. Plant Cell 27, 1999–2015. doi: 10.1105/tpc.15.00135 26139346 PMC4531349

[B184] Schneider H. M. (2022). Characterization, costs, cues and future perspectives of phenotypic plasticity. Ann. Bot. 130, 131–148. doi: 10.1093/aob/mcac087 35771883 PMC9445595

[B185] Schwartz M. F. Peters R. Hunt A. M. Abdul-Matin A. K. Van den Broeck L. Sozzani R. (2021). Divide and conquer: The initiation and proliferation of meristems. Crit. Rev. Plant Sci. 40, 147–156. doi: 10.1080/07352689.2021.1915228 37339054

[B186] Sequeira-Mendes J. Gutierrez C. (2015). Links between genome replication and chromatin landscapes. Plant J. For. Cell. Mol. Biol. 83, 38–51. doi: 10.1111/tpj.12847 25847096

[B187] Séraphin B. (1995). Sm and Sm-like proteins belong to a large family: Identification of proteins of the U6 as well as the U1, U2, U4 and U5 snRNPs. EMBO J. 14, 2089–2098. doi: 10.1002/j.1460-2075.1995.tb07200.x 7744014 PMC398309

[B188] Shen F. Hu C. Huang X. He H. Yang D. Zhao J. . (2023). Advances in alternative splicing identification: Deep learning and pantranscriptome. Front. Plant Sci. 14, 1232466. doi: 10.3389/fpls.2023.1232466 37790793 PMC10544900

[B189] Shi F. Cai F. F. Cai L. Lin X. Y. Zhang W. Wang Q. Q. . (2017). Overexpression of SYF2 promotes cell proliferation and correlates with poor prognosis in human breast cancer. Oncotarget 8, 88453–88463. doi: 10.18632/oncotarget.18188 29179448 PMC5687618

[B190] Shimotohno A. Aki S. S. Takahashi N. Umeda M. (2021). Regulation of the plant cell cycle in response to hormones and the environment. Annu. Rev. Plant Biol. 72, 273–296. doi: 10.1146/annurev-arplant-080720-103739 33689401

[B191] Shin C. Manley J. L. (2002). The SR protein SRp38 represses splicing in M phase cells. Cell 111, 407–417. doi: 10.1016/S0092-8674(02)01038-3 12419250

[B192] Shinriki S. Hirayama M. Nagamachi A. Yokoyama A. Kawamura T. Kanai A. . (2022). DDX41 coordinates RNA splicing and transcriptional elongation to prevent DNA replication stress in hematopoietic cells. Leukemia 36, 2605–2620. doi: 10.1038/s41375-022-01708-9 36229594 PMC9613458

[B193] Song X. Wan X. Huang T. Zeng C. Sastry N. Wu B. . (2019). SRSF3-regulated RNA alternative splicing promotes glioblastoma tumorigenicity by affecting multiple cellular processes. Cancer Res. 79, 5288–5301. doi: 10.1158/0008-5472.CAN-19-1504 31462429 PMC6801100

[B194] Soni N. Bacete L. (2023). The interplay between cell wall integrity and cell cycle progression in plants. Plant Mol. Biol. 113, 367–382. doi: 10.1007/s11103-023-01394-w 38091166 PMC10730644

[B195] Staiger D. Brown J. W. S. (2013). Alternative splicing at the intersection of biological timing, development, and stress responses. Plant Cell 25, 3640–3656. doi: 10.1105/tpc.113.113803 24179132 PMC3877812

[B196] Strikoudis A. Lazaris C. Trimarchi T. Neto A. L. G. Yang Y. Ntziachristos P. . (2016). Regulation of transcriptional elongation in pluripotency and cell differentiation by the PHD-finger protein Phf5a. Nat. Cell Biol. 18, 1127–1138. doi: 10.1038/ncb3424 27749823 PMC5083132

[B197] Strini E. J. Bertolino L. T. San Martin J. A. B. Souza H. A. O. Pessotti F. Pinoti V. F. . (2022). Stigma/style cell-cycle inhibitor 1, a regulator of cell proliferation, interacts with a specific 14-3-3 protein and is degraded during cell division. Front. Plant Sci. 13, 857745. doi: 10.3389/fpls.2022.857745 35444668 PMC9013909

[B198] Swaraz A. M. Park Y. D. Hur Y. (2011). Knock-out mutations of Arabidopsis SmD3-b induce pleotropic phenotypes through altered transcript splicing. Plant Sci. 180, 661–671. doi: 10.1016/j.plantsci.2011.01.011 21421416

[B199] Szklarczyk D. Nastou K. Koutrouli M. Kirsch R. Mehryary F. Hachilif R. . (2025). The STRING database in 2025: protein networks with directionality of regulation. Nucleic Acids Res. 53, D730–D737. doi: 10.1093/nar/gkae1113 39558183 PMC11701646

[B200] Tan C. W. Sim D. Y. Zhen Y. Tian H. Koh J. Roca X. (2024). PRPF40A induces inclusion of exons in GC-rich regions important for human myeloid cell differentiation. Nucleic Acids Res. 52, 8800–8814. doi: 10.1093/nar/gkae557 38943321 PMC11347146

[B201] Tanaka I. Chakraborty A. Saulnier O. Benoit-Pilven C. Vacher S. Labiod D. . (2020). ZRANB2 and SYF2-mediated splicing programs converging on ECT2 are involved in breast cancer cell resistance to doxorubicin. Nucleic Acids Res. 48, 2676–2693. doi: 10.1093/nar/gkz1213 31943118 PMC7049692

[B202] Teng T. Tsai J. H. Puyang X. Seiler M. Peng S. Prajapati . (2017). Splicing modulators act at the branch point adenosine binding pocket defined by the PHF5A-SF3b complex. Nat. Commun. 8, 15522. doi: 10.1038/ncomms15522 28541300 PMC5458519

[B203] Tran J. C. Zamdborg L. Ahlf D. R. Lee J. E. Catherman A. D. Durbin K. R. . (2011). Mapping intact protein isoforms in discovery mode using top-down proteomics. Nature 480, 254–258. doi: 10.1038/nature10575 22037311 PMC3237778

[B204] Tsai Y. S. Dominguez D. Gomez S. M. Wang Z. (2015). Transcriptome-wide identification and study of cancer-specific splicing events across multiple tumors. Oncotarget 6(33), 6825–6839. doi: 10.18632/oncotarget.3145 25749525 PMC4466652

[B205] Tsukamoto T. Gearhart M. D. Kim S. Mekonnen G. Spike C. A. Greenstein D. (2020). Insights into the involvement of spliceosomal mutations in myelodysplastic disorders from analysis of SACY-1/DDX41 in Caenorhabditis elegans. Genetics 214, 869–893. doi: 10.1534/genetics.119.302973 32060018 PMC7153925

[B206] Tyagi P. Singh D. Mathur S. Singh A. Ranjan R. (2022). Upcoming progress of transcriptomics studies on plants: An overview. Front. Plant Sci. 13, 1030890. doi: 10.3389/fpls.2022.1030890 36589087 PMC9798009

[B207] Ullah F. Hamilton M. Reddy A. S. N. Ben-Hur A. (2018). Exploring the relationship between intron retention and chromatin accessibility in plants. BMC Genomics 19, 21. doi: 10.1186/s12864-017-4393-z 29304739 PMC5756433

[B208] Vandepoele K. Raes J. De Veylder L. Rouzé P. Rombauts S. Inzé D. (2002). Genome-wide analysis of core cell cycle genes in Arabidopsis. Plant Cell 14, 903–916. doi: 10.1105/tpc.010445 11971144 PMC150691

[B209] Van Leuken R. J. Luna-Vargas M. P. Sixma T. K. Wolthuis R. M. Medema R. H. (2008). Usp39 is essential for mitotic spindle checkpoint integrity and controls mRNA-levels of aurora B. Cell. Cycle (Georgetown Tex.) 7, 2710–2719. doi: 10.4161/cc.7.17.6553 18728397

[B210] Velappan Y. Signorelli S. Considine M. J. (2017). Cell cycle arrest in plants: what distinguishes quiescence, dormancy and differentiated G1? Ann. Bot. 120, 495–509. doi: 10.1093/aob/mcx082 28981580 PMC5737280

[B211] Vivek Hari Sundar G. Madhu A. Archana A. Shivaprasad P. V. (2024). Plant histone variants at the nexus of chromatin readouts, stress and development. Biochim. Biophys. Acta (BBA) - Gen. Subj. 1868, 130539. doi: 10.1016/j.bbagen.2023.130539 38072208

[B212] Wang B. B. Brendel V. (2006). Molecular characterization and phylogeny of U2AF35 homologs in plants. Plant Physiol. 140, 624–636. doi: 10.1104/pp.105.073858 16407443 PMC1361329

[B213] Weinreb J. T. Gupta V. Sharvit E. Weil R. Bowman T. V. (2022). Ddx41 inhibition of DNA damage signaling permits erythroid progenitor expansion in zebrafish. Haematologica 107, 644–654. doi: 10.3324/haematol.2020.257246 33763998 PMC8883538

[B214] Wood K. A. Ellingford J. M. Thomas H. B. Genomics UK Research Consortium Douzgou S. Beaman G. M . (2022). Expanding the genotypic spectrum of TXNL4A variants in Burn-McKeown syndrome. Clin. Genet. 101, 255–259. doi: 10.1111/cge.14082 34713892

[B215] Xin X. Wang S. Pan Y. Ye L. Zhai T. Gu M. . (2024). MYB transcription factor CDC5 activates CBF3 expression to positively regulates freezing tolerance via cooperating with ICE1 and histone modification in Arabidopsis. Plant Cell. Environ. 48, 97–108. doi: 10.1111/pce.15144 39248548

[B216] Xin X. Ye L. Zhai T. Wang S. Pan Y. Qu K. . (2025). CELL DIVISION CYCLE 5 controls floral transition by regulating flowering gene transcription and splicing in Arabidopsis. Plant Physiol. 197. doi: 10.1093/plphys/kiae616 39560102

[B217] Xiong F. Ren J. J. Yu Q. Wang Y. Y. Lu C. C. Kong L. J. . (2019). AtU2AF65b functions in abscisic acid mediated flowering via regulating the precursor messenger RNA splicing of ABI5 and FLC in Arabidopsis. New Phytol. 223, 277–292. doi: 10.1111/nph.15756 30790290

[B218] Xu N. Ren Y. Bao Y. Shen X. Kang J. Wang N. . (2023). PUF60 promotes cell cycle and lung cancer progression by regulating alternative splicing of CDC25C. Cell Rep. 42, 113041. doi: 10.1016/j.celrep.2023.113041 37682709 PMC13034450

[B219] Xu F. Xu S. Wiermer M. Zhang Y. Li X. (2012). The cyclin L homolog MOS12 and the MOS4-associated complex are required for the proper splicing of plant resistance genes. Plant Journal: For. Cell. Mol. Biol. 70, 916–928. doi: 10.1111/j.1365-313X.2012.04906.x 22248079

[B220] Xu W. Cao M. Zheng H. Tan X. Li L. Cui G. . (2014). Upregulation of SYF2 is associated with neuronal apoptosis caused by reactive astrogliosis to neuroinflammation. Journal of neuroscience research, 92(3), 318–328. doi: 10.1002/jnr.23312 24301298

[B221] Yan S. Bhawal R. Yin Z. Thannhauser T. W. Zhang S. (2022). Recent advances in proteomics and metabolomics in plants. Mol. Hortic. 2, 17. doi: 10.1186/s43897-022-00038-9 37789425 PMC10514990

[B222] Yan Q. Xia X. Sun Z. Fang Y. (2017). Depletion of Arabidopsis SC35 and SC35-like serine/arginine-rich proteins affects the transcription and splicing of a subset of genes. PloS Genet. 13, e1006663. doi: 10.1371/journal.pgen.1006663 28273088 PMC5362245

[B223] Ye J. Beetz N. O'Keeffe S. Tapia J. C. Macpherson L. Chen W. V. . (2015). hnRNP U protein is required for normal pre-mRNA splicing and postnatal heart development and function. PNAS 112, E3020–E3029. doi: 10.1073/pnas.1508461112 26039991 PMC4466706

[B224] Yeh P. C. Yeh C. C. Chen Y. C. Juang Y. L. (2012). RED, a spindle pole-associated protein, is required for kinetochore localization of MAD1, mitotic progression, and activation of the spindle assembly checkpoint. J. Biol. Chem. 287, 11704–11716. doi: 10.1074/jbc.M111.299131 22351768 PMC3320919

[B225] Yi D. Alvim Kamei C. L. Cools T. Vanderauwera S. Takahashi N. Okushima Y. . (2014). The Arabidopsis SIAMESE-RELATED cyclin-dependent kinase inhibitors SMR5 and SMR7 regulate the DNA damage checkpoint in response to reactive oxygen species. Plant Cell 26, 296–309. doi: 10.1105/tpc.113.118943 24399300 PMC3963576

[B226] Yin R. Xia K. Xu X . (2023). Spatial transcriptomics drives a new era in plant research. The Plant journal : for cell and molecular biology, 116(6), 1571–1581. doi: 10.1111/tpj.16437 37651723

[B227] Yoshiyama K. O. (2015). SOG1: a master regulator of the DNA damage response in plants. Genes Genet. Syst. 90, 209–216. doi: 10.1266/ggs.15-00011 26617076

[B228] Yoshiyama K. Conklin P. A. Huefner N. D. Britt A. B. (2009). Suppressor of gamma response 1 (SOG1) encodes a putative transcription factor governing multiple responses to DNA damage. PNAS 106, 12843–12848. doi: 10.1073/pnas.0810304106 19549833 PMC2722309

[B229] Yoshiyama K. O. Kimura S. Maki H. Britt A. B. Umeda M. (2014). The role of SOG1, a plant-specific transcriptional regulator, in the DNA damage response. Plant Signaling Behav. 9, e28889. doi: 10.4161/psb.28889 24736489 PMC4091597

[B230] Yoshiyama K. O. Kobayashi J. Ogita N. Ueda M. Kimura S. Maki H. . (2013). ATM-mediated phosphorylation of SOG1 is essential for the DNA damage response in Arabidopsis. EMBO Rep. 14, 817–822. doi: 10.1038/embor.2013.112 23907539 PMC3790055

[B231] Yu S. Han J.-H. Chhoeun C. Lee B. (2016). Genetic screening for Arabidopsis mutants defective in STA1 regulation under thermal stress implicates the existence of regulators of its specific expression, and the genetic interactions in the stress signaling pathways. Front. Plant Sci. 7. doi: 10.3389/fpls.2016.00618 27242824 PMC4861721

[B232] Yuan X. Sun X. Shi X. Jiang C. Yu D. Zhang W. . (2015). USP39 promotes the growth of human hepatocellular carcinoma *in vitro* and *in vivo*. Oncol. Rep. 34, 823–832. doi: 10.3892/or.2015.4065 26081192

[B233] Yuan J. Xu B. Su Y. Zhang P. Zhang X. Gong P. (2025). Identification of USP39 as a prognostic and predictive biomarker for determining the response to immunotherapy in pancreatic cancer. BMC Cancer 25, 758. doi: 10.1186/s12885-025-14096-x 40264098 PMC12016207

[B234] Zhang N. Kaur R. Akhter S. Legerski R. J. (2009). Cdc5L interacts with ATR and is required for the S-phase cell-cycle checkpoint. EMBO Rep. 10, 1029–1035. doi: 10.1038/embor.2009.122 19633697 PMC2750050

[B235] Zhang Y. Li Z. Chen N. Huang Y. Huang S. (2020). Phase separation of Arabidopsis EMB1579 controls transcription, mRNA splicing, and development. PloS Biol. 18, e3000782. doi: 10.1371/journal.pbio.3000782 32692742 PMC7413564

[B236] Zhang Z. Liu W. Bao X. Sun T. Wang J. Li M. . (2022). USP39 facilitates breast cancer cell proliferation through stabilization of FOXM1. American journal of cancer research, 12(8), 3644–3661. 36119839 PMC9442023

[B237] Zhang S. Shi W. Chen Y. Xu Z. Zhu J. Zhang T. . (2015). Overexpression of SYF2 correlates with enhanced cell growth and poor prognosis in human hepatocellular carcinoma. Mol. Cell. Biochem. 410, 1–9. doi: 10.1007/s11010-015-2533-9 26260052

[B238] Zhang Z. Zhang S. Zhang Y. Wang X. Li D. Li Q. . (2011). Arabidopsis floral initiator SKB1 confers high salt tolerance by regulating transcription and pre-mRNA splicing through altering histone H4R3 and small nuclear ribonucleoprotein LSM4 methylation. Plant Cell 23, 396–411. doi: 10.1105/tpc.110.081356 21258002 PMC3051234

[B239] Zhao L. Li Y. Xie Q. Wu Y. (2017). Loss of CDKC;2 increases both cell division and drought tolerance in Arabidopsis thaliana. Plant Journal: For. Cell. Mol. Biol. 91, 816–828. doi: 10.1111/tpj.13609 28622431

[B240] Zhou Z. Fu X. D. (2013). Regulation of splicing by SR proteins and SR protein-specific kinases. Chromosoma 122, 191–207. doi: 10.1007/s00412-013-0407-z 23525660 PMC3660409

[B241] Zhou Z. Liu Y. Nie X. Cao J. Zhu X. Yao L. . (2014). Involvement of upregulated SYF2 in Schwann cell differentiation and migration after sciatic nerve crush. Cell. Mol. Neurobiol. 34, 1023–1036. doi: 10.1007/s10571-014-0078-1 24962097 PMC11488921

[B242] Zhu F. Wen W. Cheng Y. Alseekh S. Fernie A. R. (2023). Integrating multiomics data accelerates elucidation of plant primary and secondary metabolic pathways. aBIOTECH 4, 47–56. doi: 10.1007/s42994-022-00091-4 37220537 PMC10199974

